# Novel antimicrobial biodegradable composite films as packaging materials based on shellac/chitosan, and ZnAl_2_O_4_ or CuAl_2_O_4_ spinel nanoparticles

**DOI:** 10.1038/s41598-024-78261-1

**Published:** 2024-11-13

**Authors:** Salah A. A. Mohamed, Saleh D. Mekkey, Abdelmageed M. Othman, Mohamed El-Sakhawy

**Affiliations:** 1 Packaging Materials Department, National Research Centre, 33 El-Bohouth Str., Dokki, P.O. 12622, Giza, Egypt; 2 Chemistry Department, Faculty of Science, Al-Azhar University, Nasr City, 11884 Cairo Egypt; 3 Microbial Chemistry Department, Biotechnology Research Institute, National Research Centre, 33 El Bohouth St., Dokki, Giza, 12622 Egypt; 4 Faculty of Biotechnology, German International University, New Administrative Capital, Cairo Egypt; 5 Cellulose and Paper Department, National Research Centre, 33 El-Bohouth Str., Dokki, P.O. 12622, Giza, Egypt

**Keywords:** Chitosan, Shellac, Spinel materials, ZnAl_2_O_4_, CuAl_2_O_4_, Nanoparticle, Packaging materials, Biological techniques, Chemistry, Materials science, Nanoscience and technology

## Abstract

ZnAl_2_O_4_ and CuAl_2_O_4_ spinel nanoparticles were prepared by a modified Pechini method and used with the natural chitosan (CS) and shellac (SH) polymers to form novel composite membranes as promising food packaging materials. The selection of ZnAl_2_O_4_ and CuAl_2_O_4 _spinel nanoparticles was based on their antibacterial characteristics, availability, and economy. Using a straightforward and adaptable solution mixing and casting method, the bio-composites were created. The mechanical, physical, antibacterial, homogeneity and air permeability properties of composite films were investigated. The film structure was evaluated in terms of component interactions using FTIR spectra. The addition of 10% SH increased the tensile strength, percentage strain at maximum load, Young’s modulus, and burst strength by 114–101%, 3.6–8.4, 103–119, and 179–153% for low and middle M.wt./CS respectively. Chitosan/shellac-CuAl_2_O_4_ composite has superior properties compared to ZnAl_2_O_4_ composite. In general, 0.05% spinel provides a composite having better qualities than that of 0.1 additions. Middle M.wt. chitosan provides a composite with superior properties compared to that of low M.wt. The incorporation of ZnAl_2_O_4_ or CuAl_2_O_4_ enhanced the thermal stability of the SH/CS composite. ZnAl_2_O_4_ provides superior thermal stability than CuAl_2_O_4_. When shellac/CS film structure is treated with the previously indicated ZnAl_2_O_4_ or CuAl_2_O_4_ formulation, the % swelling decreases along with an increasing in the gel fraction. The antimicrobial assessment using inhibition zone diameter and shake flask methods showed that a composite of 1:9 shellac/chitosan/0.05% of CuAl_2_O_4_ exerted the highest Gram-positive antibacterial activity against *B. mycoides* (21 mm), and *C. albicans* (22 mm). So, these enhancements make chitosan/shellac/ZnAl_2_O_4_ or CuAl_2_O_4 _composite films a good alternative to producing food packaging materials.

## Introduction

Worldwide, the food packaging industry makes up over 2% of the goods produced in industrialized nations. The food industry searches for innovative, affordable, and environmentally responsible packaging materials to preserve and track food quality^1–3^. In recent decades, researchers have been interested in finding alternatives to synthetic food packaging polymers to avoid severe environmental risks. This is due to the fact that their disposal methods are not suitable and they are non-biodegradable^4^. Therefore, the researchers were interested in finding environmentally friendly, safe, and non-toxic alternatives, such as biopolymers, as they are biodegradable, making them suitable as an alternative to industrial plastic films.

Enhancing the biodegradable film’s mechanical strength, thermal stability, and barrier effectiveness can be done in several ways. Adding strengthening nanofillers to natural materials such as chitosan, HPMC, CMC, and starch matrix is one of them. Additionally, under these conditions, nanocomposite films can operate as a transporters of certain active compounds, including as antioxidants, bacteriostatic agents, plant extracts, and enzymes, to improve food safety and extend the shelf life of food products^1,2^. To create materials with intriguing qualities suitable for food packaging applications, the researchers tried adding the most popular inorganic/mineral nanoparticles and mineral oxides, such as silver, copper, titanium oxide, zinc oxide, magnesium, calcium oxide, etc.^5,6^. As a result, they are also more stable in harsh environments like high pressure and temperature. Some of them have even been shown to be non-toxic and to include essential mineral elements for human health. Thus, in the food packaging business, bio-nanoparticles have been noted as a possible substitute^4,6^. The important role of metal oxide nanomaterials like AgCl–TiO_2_^7^, ZnO^8,9^, and TiO_2_^10^ in the fabrication of nanocomposite films coupling the polymer matrices with chitosan, PVC, and carboxymethyl cellulose is highlighted by the recent studies on metal oxide nanocomposite-based food packaging. Composites contain nanoparticles have enhanced mechanical qualities and high-water permeability or hydrophilicity. Additionally, by inducing cellular material leakage via the damaged membrane, the TiO_2_ and ZnO nano-compounds have shown action against both Gram-positive and Gram-negative bacteria^8,11^. Similarly, under visible light radiation, TiO_2_ and Ag-doped TiO_2_ nanoparticles have been shown to exhibit photocatalytic antibacterial ability^12,13^.

Chitosan and shellac are natural polymers used in many fields such as food packaging technology and the pharmaceutical industry^14–16^. The non-toxic, biodegradable, biocompatible, and antibacterial qualities of chitosan make it an invaluable tool in the biotechnology, food, and medical industries. This polymer’s polycationic properties make it useful in the chemical, textile, membrane, and wastewater treatment sectors^17–21^. Furthermore, the commercial uses of chitosan in paper manufacturing, tissue engineering, food safety, cosmetics, and agriculture have drawn a lot of interest^20–23^, and it’s an excellent biomaterial used in wound dressing^24,25^. Aquercetin-loaded nanoencapsulation using zein, shellac, and chitosan was created to provide a promising encapsulation approach for bioactive hydrophobic compounds that are unstable^26,27^. As for shellac’s interesting properties, such as low acid permeability, low water permeability, gloss, natural origin, biocompatibility, and biodegradation, it has been used in pharmaceutical and agricultural products, sweets, and foodstuffs^28^. Shellac is utilized in pharmaceutical applications as an enteric coating for oral medication administration^16,29–31^. Shellac has a potential for use in the food industry overall^28^. To prolong shellac’s use in packaging applications, shellac has undergone both physical and chemical modifications. Lastly, a comparison between shellac and common crude-based polymers and conventional bio-based materials is made to examine the non-toxic and biodegradable properties of shellac as well as its packaging possibilities^32^.

Attractive classes of mixed oxide ceramics with significant technological implications are called spinel materials. AB_2_O_4_ is the general formula for these compounds, where A and B are divalent and trivalent metal cations that typically occupy a cubic lattice as tetrahedral and octahedral sites respectively^33^. ZnAl_2_O_4_ and CuAl_2_O_4_ spinels are two important species in this family which are often hydrophobic, mechanically robust, chemically and thermally stable, and have low surface acidity. Spinel is used as catalyst support as it is a stable substance that is highly resistant to alkalis and acids. Furthermore, the melting values of this kind of material are high^26,29^. Zinc aluminate is known for its wide energy band gap, high chemical and thermal stability, high fluorescence efficiency, improved mechanical strength, anti-reflection window layer coating in solar cells, superior optical transmittance, gas sensors, low surface acidity, and high photocatalytic properties. All these properties have made zinc aluminate superior for use in the field of photovoltaic devices, electroluminescent displays, optical coatings, high-efficiency phosphors, catalysts, packaging, etc.^34^. Copper aluminate spinel (CuAl_2_O_4_) is also known to be active in the decomposition of some organic compounds, reduction of nitrogen dioxide, and oxidation of alcohols. They also have many applications such as sensor materials and ceramic pigments^34,35^.

Additionally, it is known that copper and zinc aluminate spinel are active in the breakdown of various organic molecules^34,36^. It was also used in the study of the adsorption of some organic dyes, which harm the environment^37^ and it can be used as an antibacterial material^38–40^. The preparation of metal aluminates spinel has been obtained by several techniques, including solid-state reaction^41^, sol–gel^42–44^, co-precipitation method^45,46^, modified citrate^47^, combustion^48^ polymeric precursor^33^, hydrothermal^49^, microwave-hydrothermal^50^ and Pechini method^51–53^. The Pechini technique has the foremost advantages, the manipulation easiness and the fact that it is not sensitive to the water presence, known to be simple, cost-effective, and versatile. Which makes it simpler than the sol–gel technique based on metal alkoxides^37^.

Despite the special benefits of chitosan (CS) film, its poor physicochemical qualities severely restrict its practical use as it is highly hydrophilic and has low thermal stability. Spinel metal oxides are important classes of inorganic materials, and they are non-toxic and inexpensive and have been widely used in modern industry. Spinel aluminates show advantages of resistance against acids and alkalis, excellent hardness, and high-temperature stabilities, which have varied vital applications. It’s interesting to note that CS films’ qualities could be improved by using shellac and ZnAl_2_O_4_ or CuAl_2_O_4_ spinel nanoparticles which were based on their thermal stability, hydrophobicity, antibacterial characteristics, availability, and economy instead of non-biodegradable synthetic plastic polymer. So, this research aims to prepare ZnAl_2_O_4_ and CuAl_2_O_4_ nanoparticles by a modified Pechini method, to work with chitosan and shellac polymers as new composite membranes, and study their mechanical, physical, elongation, homogeneity, air permeability, and antimicrobial properties, to see its suitability for use as a promising food packaging material.

## Experimental

### Materials and methods

#### Materials

Chitosan (CS) Deacetylated chitin, poly (d-glucosamine), CS1 middle molecular weight: M.wt.100,000–300,000 Da & CS2, low molecular weight: M.wt. 35,000–50,000 Da, was purchased from ACROS, ORGANICS, New Jersey, USA. Shellac was a commercial grade and used as received. Pure analytical grades of aluminum chloride (AlCl_3_), zinc acetate (Zn(CH_3_COO)_2_·2H_2_O), cupric chloride (CuCl_2_·2H_2_O), and other chemicals were purchased from Sigma-Aldrich ChemieGmbh, Germany, and used as received. Microbial cultures used for the assessment of antimicrobial activity (*Bacillus mycoides*, *Escherichia coli*, and *Candida albicans*) were obtained from the culture collection of the Microbial Chemistry Department, National Research Centre, Egypt. The microbial strains were grown on nutrient agar medium (70148-Nutrient agar) that was purchased from Fluka, Spain.

#### Methods

##### Modified Pechini method

Aluminum chloride was added to zinc acetate or cupric chloride with the mole ratio of Al^3+^: M^2+^ = 2:1 (M: Zn^2+^ or Cu^2+^) to prepare ZnAl_2_O_4_ or CuAl_2_O_4_ powder, respectively. Citric acid (CA) was added after the mixture was dissolved in distilled water (molar ratio CA: total cations 1:1). The mixture was stirred until a transparent, yellowish solution formed. This solution was supplemented with ethylene glycol (EG, M.Wt = 62.07) in the molar ratio of 2:1 between EG and CA. At about 80 °C, the solution was constantly agitated to hasten the evaporation of surplus water and boost the polyesterification process rate. There was no turbidity or precipitation during the polyesterification process. Once viscous gel was obtained, the process was terminated. After that, the gel was dried for 24 h in an oven at 150 °C. The xerogels were milled after calcination at 800 °C, for 2 or 6 h to attain CuAl_2_O_4_ or ZnAl_2_O_4_ powder, respectively^37^.

##### Preparation of composite films

A final concentration of 5% (g/V) was achieved by dissolving 5 g of ground shellac in 100 mL of ethanol at room temperature. The solution was stirred for roughly an hour. Undissolved shellac portion and contaminations were removed via filtration. Chitosan, low and middle molecular weight, was dissolved in 1% acetic acid to create solutions with 2% weight percent concentrations. Chitosan was dissolved in 1% acetic acid to create solutions with 2% weight percent concentrations at 55 °C using mechanical stirring for 2 h to get a completely translucent mixture. After that, wait for 24 h to get a transparent solution. To formulate shellac/chitosan composites, different ratios of chitosan low M.wt. were blended with shellac (10:90 and 20:80%), in the case of chitosan middle M.wt. one ratio was used with shellac (10:90%) at room temperature for 1 h of the aforementioned prepared shellac and chitosan solutions as well as 25% of glycerol, based on the SH/CS blend solid content (w/w), as a plasticizer, were mixed with stirring followed by sonication to remove the air bubbles. The solution-casting method was used to create the shellac/chitosan films then dried at 45 °C in an air oven dry^54^. For the loading of ZnAl_2_O_4_-NPs and CuAl_2_O_4_-NPs, the composite films were prepared as above and different weights of ZnAl_2_O_4_-NPs or CuAl_2_O_4_**-**NPs (0, 0.05, and 0.1%) were added and left for 2 h with stirring. The prepared composite film compositions and code are listed in Table 1**.**

**Table 1:** The mechanical properties of chitosan/shellac composite films.

Designa-tion of film	Reaction conditions			TH	Swelling (%)	Gel fraction	Percentage strain at maximum load	AR Sec	Biodegrada-tion (%)
	SH 5%	CH LM 2%	CH MM 2%						
SS_zero_	00	100	00	0.26 ± 0.01	7.03 ± 1.12	50.61 ± 0.52	45.13 ± 2.93	Nil	52.90 ± 0.64
SSa	10	90	00	0.25 ± 0.03	13.95 ± 1.39	58.95 ± 1.20	46.77 ± 0.35	Nil	57.09 ± 0.52
SSb	20	80	00	0.22 ± 0.01	2.52 ± 0.33	53.68 ± 2.32	10.67 ± 3.09	Nil	46.99 ± 0.35
SSc	00	00	100	0.25 ± 0.03	6.52 ± 1.12	51.68 ± 0.56	32.11 ± 2.10	Nil	49.12 ± 0.25
SSd	10	00	90	0.24 ± 0.01	15.63 ± 1.31	59.58 ± 0.68	29.40 ± 4.66	Nil	39.17 ± 0.21

*SH* Shellac, *CH* Chitosan, *LM* Low M.wt, *MM* Middle M.wt., *TH* Thickness, *AR* Air permeability.

#### Characterization

##### X-ray diffraction pattern (XRD)

The specimens were cut into a rectangular strip measuring 2 × 4 cm. A Philips diffractometer (type PW-3710) was used to measure the X-ray diffraction pattern (XRD) at room temperature. Ni-filtered copper radiation (θ = 1.5404 Å) was used to run the patterns at 30 kV, 10 mA, and a scanning speed of 2 θ = 2.5°/min. Using the Debye–Scherrer Eq. [(Kλ)/(βcosƟ)], where K is a constant equal to 0.9, λ is the wavelength of the Cu Kα radiation, and β is the half peak width of the diffraction peak in radiant, the mean particle size was determined^37^.

##### FT-IR

Samples were cut into circles with a diameter of 3 mm. Using the Fourier Transform Infrared (FTIR) Spectroscopy, FT-IR JASCO 4100 Spectrometer, the specimens’ infrared spectra were captured. This test was run to examine how the functional group of the coated and untreated specimens had changed. The wavenumber range for the spectra was 4000–500 cm^−1^^55^.

##### Scanning electron microscope

Using an acceleration voltage of 20 kV, FEI Quanta 200 scanning electron microscope (FEI Company BV, Netherlands) was used to image the microstructure of coated paper sheets^56^.

##### Thermal analysis (TGA/DTG)

Thermal gravimetric analysis (TGA) was used to investigate the thermal stability of composite film samples. The weight loss of the composite film samples was calculated using the Shimadzu TGA-50 instrument as a function of temperature. Differential thermogravimetry, or DTG, is a TGA derivative that calculates the rate at which weight varies with temperature. It is useful for determining the temperature at which a substance undergoes a certain thermal event, such as combustion or breakdown. The samples typically ranged in mass from 11 to 34 mg. Under an inert nitrogen atmosphere, the test was run at a flow rate of 30 ml/min, a heating rate of 10 °C/min, and a temperature range of 30–1000 °C^57,58^.

##### Thickness

The specimens’ thickness was measured using a Mitutoyo “Absolute AosDigimatic” measuring range of 0–150 mm, model no. CD-15APX. An average of five measurements was taken.

##### Tensile strength

Before testing, the films were conditioned for 48 h at 50% relative humidity. Tensile strength qualities were evaluated using a Lloyd instrument (Lloyd Instruments, West Sussex, United Kingdom) equipped with a 100-N load cell at 25 °C. The samples were strips of 10 mm in width and 15 cm in length, with a crosshead speed of 2 mm/min. Every sample was measured five times, and the resulting average^59^.

##### Burst strength

The burst strength of both untreated and treated specimens was used to assess their resistance to bursting following ISO 2758^60^.

##### Air permeability

The Anderson & Sorensen Model 5 (No. 11772), Bendtsen Smoothness and Porosity Tester D207490, Denmark, was used to measure the air permeability^60^.

##### The degree of swelling and gel fraction analysis

The degree of swelling (SW) was performed by steeping films or treated fabric samples in distilled water at 37 °C and pH 7 for 24 h followed by removing the samples from water, wiping gently with a filter paper, and weighing. The degree of swelling percentage was evaluated according to Eq. (1):

1 $${\text{SW }}\left( \% \right) \, = \, \left( {{\text{Wh}} - {\text{Wd}}} \right)/ \, \left( {{\text{Wd}}} \right) \, \times 100$$

where Wh is the hydrated weight of the films or treated fabric samples after leaving it to swell in the distilled water whereas Wd is the dry weight of those samples. The gel fraction (GF) is an indication of the degree of firmness of the prepared films or treated fabric structures. The gel fraction percentage was assessed according to Eq. (2):

2 $${\text{GF }}\left( \% \right) \, = \, \left( {{\text{Wa}}/{\text{Wi}}} \right) \, \times 100$$

where Wa is the constant weight of the dried films or treated fabric after allowing it to swell in water with a pH of 7 at 37 °C for 24 h whereas Wi is the starting weight of the films or treated fabric^61–65^.

##### UV visible spectrophotometer

After being cut into a rectangular strip measuring 1 × 4 cm, the film was put into the UV visible spectrophotometer, JASCO V-730 spectrophotometer, at 200–800 nm, and the transmittance was measured.

##### Biodegradation characterization

According to previous reports^55,66^, the nanocomposite-based biomaterials are degraded naturally in soil. After eliminating all inert elements, soil from the uppermost layer was taken to make a homogenous mass of dirt. The average pH of 7.5 and the matching percentages of sand, silt, and clay (48.2, 38.2, and 13.8%) were found in the texture of the experimental soil. The concentrations of Ca, Mg, Na, SO_4_, and HCO_3_ ions were 2.1, 0.7, 1.3, 3.6, and 1.4 meq/l, respectively. Within a plastic container, around 100 g of earth were positioned at a depth of 3 cm. The ready samples were weighed exactly and dried for 24 h at 50 °C. They were then buried in the pots one centimeter below the surface. To keep the moisture content stable, water was sprayed once a day. The samples totally degraded after a week, and the percentage of biodegradation was ascertained.

3 $$\text{Biodegradation \% }=\frac{\text{Wi}-\text{Wf}}{\text{Wi}} \times 100$$

where $\text{Wi}$ and $\text{Wi}$ are the initial and final weights (g), respectively^55^.

##### Microorganisms and media

*Bacillus mycoides*, *Escherichia coli*, and *Candida albicans* were used as Gram-positive, Gram-negative, and non-filamentous fungal strains, respectively, to investigate the antibacterial activity of shellac, chitosan, and spinel sheets. *C. albicans*, *E. coli*, and *B. mycoides* were cultured and kept on slants of modified nutritional agar media. The adjusted medium contained the following components (g/L): 0.5 glucose; 0.25 sodium chloride; 3.0 peptone; 1.5 yeast extract; 1.5 meat extract; and 20.0 agar. The pH was brought down to 7.0 prior to autoclaving. Once the injected microbial cultures had reached maximum growth, they were kept in agar slants at 4 °C. For antimicrobial assessment utilizing the agar diffusion technique at 37 °C, the employed microbial strains were seeded and grown on nutrient agar medium (70148 Nutrient agar, Fluka, Spain) with the following components (g/L): Meat extract (1.0 g), yeast extract (2.0 g), peptone (5.0 g), sodium chloride (5.0 g), and agar (15.0 g) were added. After suspending 28 g of the ready medium in 1.0 L of distilled water, the pH of the nutrient agar medium was brought to 7.0. Subsequently, the medium was sterilized by boiling it until it completely dissolved and autoclaving it for 15 min. at 121 °C and 1.5 atmospheres. Every chemical used was of the highest purity and analytical grade.

##### Antimicrobial activity determination using the Agar diffusion technique

To evaluate the efficacy of the generated sheets as antimicrobial agents, 100 μL of re-suspended overnight culture at 37 °C (1 × 10^7^ CFU/100 μL) was added to the microbial cultures on the nutrient agar medium (70148 Nutrient agar, Fluka). Nutrient agar media was poured into Petri plates after the intended microbial inoculums were injected into the liquefied medium at approximately 45 °C. The surfaces of the firm Petri plates were covered with discs (15 mm) manufactured from various sheets after the plates were left to solidify at room temperature. Overnight, culture test plates were incubated at 37 °C. The resulting inhibitory zones were measured using the AATCC Test Method^15^.

##### Antimicrobial activity determination using the shake flask technique

The shake flask technique with the nutrient broth was also used to assess the antimicrobial activity. This approach was used to track growth inhibition caused by produced sheets. The samples were shaken for three and seventy-two hours at 37 °C in flasks, the optical absorbance changed throughout that period. Test and control sheets were cut into thin (0.3 g) pieces in triplicate and placed in sterile 250 mL Erlenmeyer flasks with 50 mL of medium. One milliliter (1 × 10^7^ CFU/100 μL) of inoculums was added to each flask. At 37 °C, the flasks were immediately shaken at 150 rpm in a rotary shaking incubator. The absorbance at 660 nm was measured after 3 and 72 h. Triplicate test results were used to assess antimicrobial effectiveness^67^.

## Results and discussion

### Preparation and characterization of ZnAl_2_O_4_-NPs and CuAl_2_O_4_-NPs

**Fig. 1:**
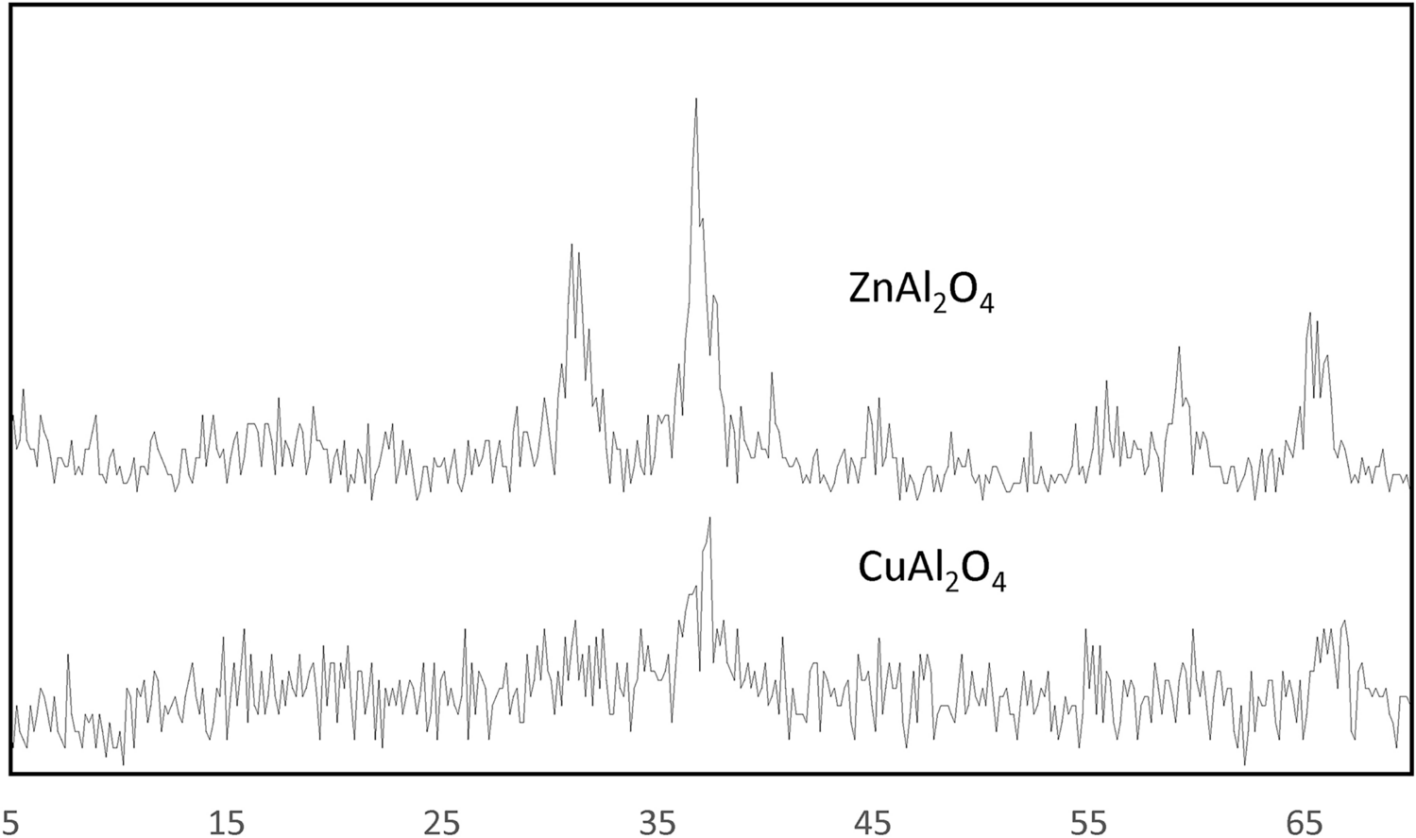
XRD of the ZnAl_2_O_4_ and CuAl_2_O_4_.

Figure 1 shows the XRD patterns of prepared samples calcined at 800 °C for 6 h for ZnAl_2_O_4_ and 2 h for CuAl_2_O_4_ (Fig. 1). For ZnAl_2_O_4_ and CuAl_2_O_4_ compounds, all diffraction peaks may be rightly indexed to face-centered cubic spinel structures. The calcined samples contain no additional crystalline phases and the ZnAl_2_O_4_ sample is more crystalline than the CuAl_2_O_4_ sample. The Scherrer formula was used to the sample’s highest intensity peak to estimate the average crystallite value. The sample was found to have an average size of 10–20 nm. As a result, the synthesis of a ZnAl_2_O_4_ and CuAl_2_O_4_ spinel during calcination is supported by XRD data.

**Fig. 2:**
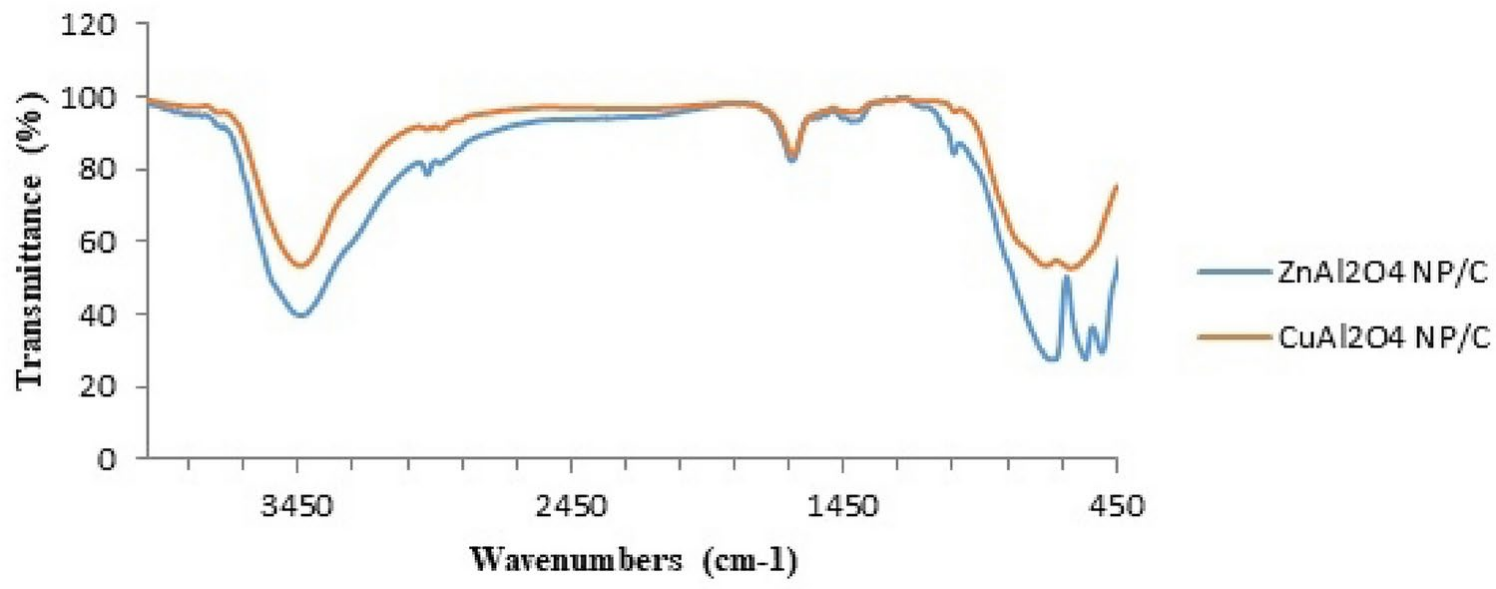
FT-IR spectra of the (**a**) ZnAl_2_O_4_ and (**b**) CuAl_2_O_4_.

Figure 2 shows the FT-IR spectra of ZnAl_2_O_4_ and CuAl_2_O_4_ (Fig. 2a,b). The absorption peak 3400–3100 cm^−1^ in both spectra was attributed to the OH^−^ stretching vibrations of the hydroxyl groups. The absorption peak at 1624 cm^−1^, which corresponded to the vibration of H–O–H in the water, was created by water adsorption during the compaction of powder specimens with KBr^4,6,10^. Figure 2a of the ZnAl_2_O_4_ compounds, exhibiting the metal-O stretching frequencies for Zn–O, Al–O, and Zn–O–Al bonds in the 500–900 cm^−1^ range^8^. The stretching frequencies of CuAl_2_O_4_ Fig. 2b are in the range of 550–850 cm^−1^ and are associated to the Cu–O, Al–O, and Cu–O–Al bond vibrations^6,7,11^. These results, which complement the XRD results, confirm the presence of the spinel crystal phase in the generated compounds.

**Fig. 3:**
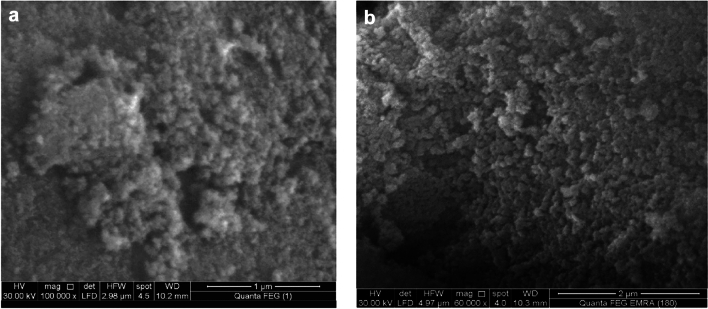
(**a**) SEM of the ZnAl_2_O_4_ spinel. (**b**) SEM of the CuAl_2_O_4_ spinel.

Figure 3a,b show the investigated SEM morphology of the ZnAl_2_O_4_ and CuAl_2_O_4_ nanostructures. In the case of the ZnAl_2_O_4_ sample, the generated nano-sized particles are well dispersed and spherical, while in the case of the CuAl_2_O_4_ sample, a little agglomerates and irregular morphology have been noticed.

### Preparation and characterization of chitosan (middle or low M.wt.)/shellac composite films

#### Mechanical study

**Fig. 4:**
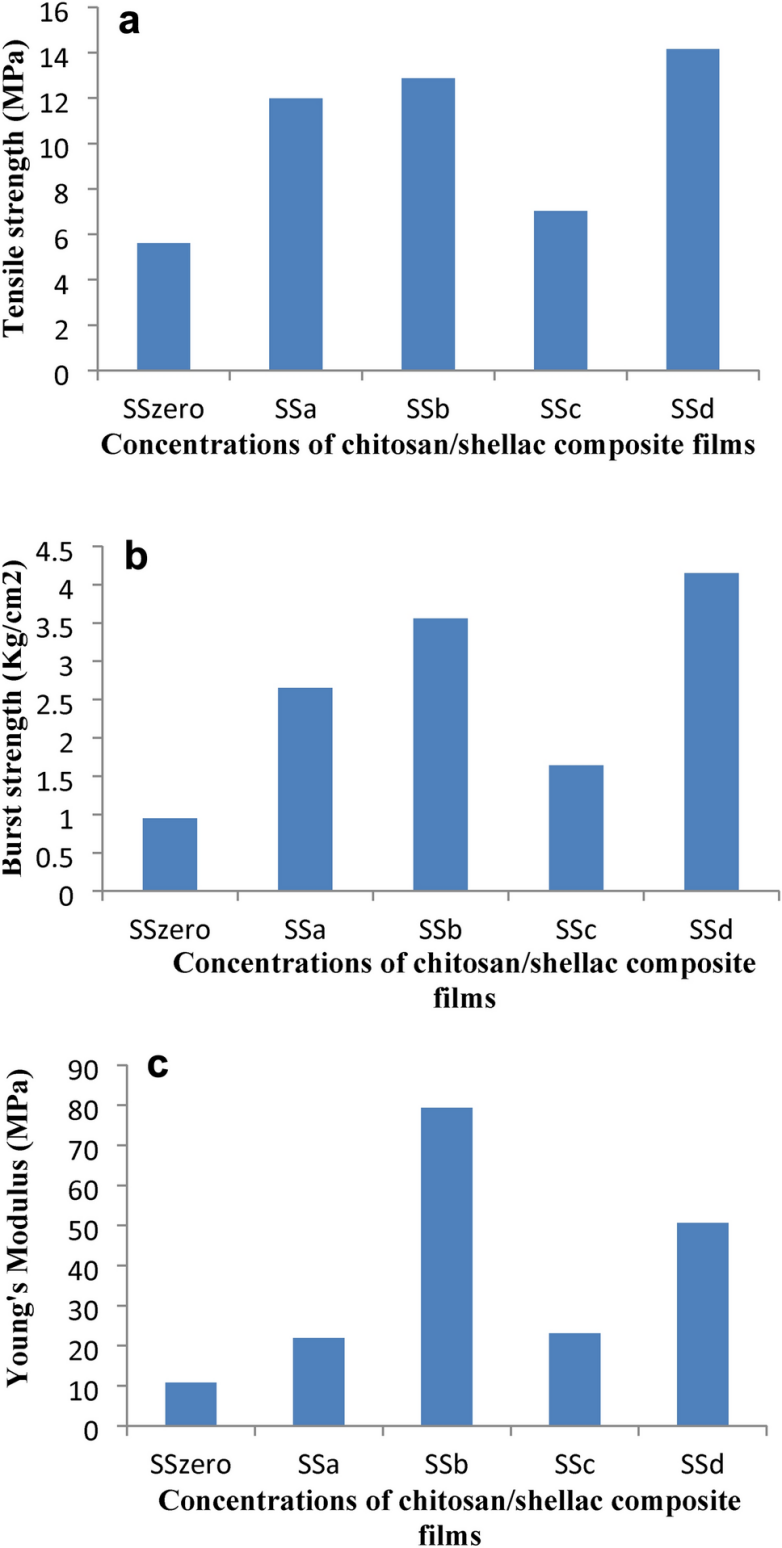
(**a**) Tensile strength of Shellac/Chitosan composite films. (**b**) Burst strength of Shellac/Chitosan composite films. (**c**) Young’s Modulus of Shellac/Chitosan composite films.

Chitosan (CS) film has proven advantages such as biodegradability, edible properties, non-toxicity, biocompatibility, and antibacterial properties; yet, the poor mechanical properties limit its productive application. Shellac is a lac insect’s resinous secretion; it has been certified non-toxic and authorized for use as a food additive. Shellac has been used to enhance the CS film properties. The mechanical properties of composites are important to suggest their prospective applications. The film material’s tensile strength (TS), percentage strain at maximum load, Young’s modulus (YM) and burst strength are a crucial metric for assessing their mechanical qualities; the higher the TS, percentage strain at maximum load, Young’s modulus (YM), and burst strength, the better the material’s mechanical qualities^68^. Therefore, Table 1 and Fig. 4a demonstrate the tensile strength (TS), percentage strain at maximum load, Young’s modulus (YM), burst strength, and air permeability (EB) of CS and CS/shellac composite.

Chitosan, low and middle M.wt., forms films of acceptable properties with superior properties for the middle M.wt. type. Shellac additions (10 and 20%) to low M.wt. chitosan significantly improved its properties, while the addition of shellac with a percentage of 30% or higher produced brittle films with poor properties. A comparable improvement in strength properties was noticed for both 10 and 20% shellac addition to low M.wt. CS, so shellac was added on 10% to middle M.wt. for further investigations.

The addition of 10% shellac increased the tensile strength, percentage strain at maximum load, Young’s modulus, and burst strength by 114–101%, 3.6–8.4, 103–119, and 179–153% for low and middle M.wt. CS, respectively Fig. 4c,b. Shellac particles dispersed uniformly in the films and the additional hydrogen bonding, due to the electrostatic interaction between the cationic charges of CS and the anionic charges of shellac particles, enhance the film’s mechanical properties. The air permeability was persevering excellent due to bridging gaps and filling of voids in CS films.

The addition of shellac more than 20% to chitosan gave bad films and upon 45% shellac, no film was formed. Our results were in consequence with similar reported results^54^ and H.-A.S. Tohamy, et al.^55^.

### Preparation and characterization of chitosan (low M.wt.)/shellac-ZnAl_2_O_4_-NPs or CuAl_2_O_4_-NPs composite films

#### Mechanical properties

Mechanical qualities are critical to maintaining film integrity when handling packaged food goods^69–74^. Spinel metal oxides are important classes of inorganic materials, and they are non-toxic and inexpensive and have been widely used in modern industry. Spinel aluminates show advantages of resistance against acids and alkalis, excellent hardness, and high-temperature stabilities, which have varied vital applications.

**Fig. 5:**
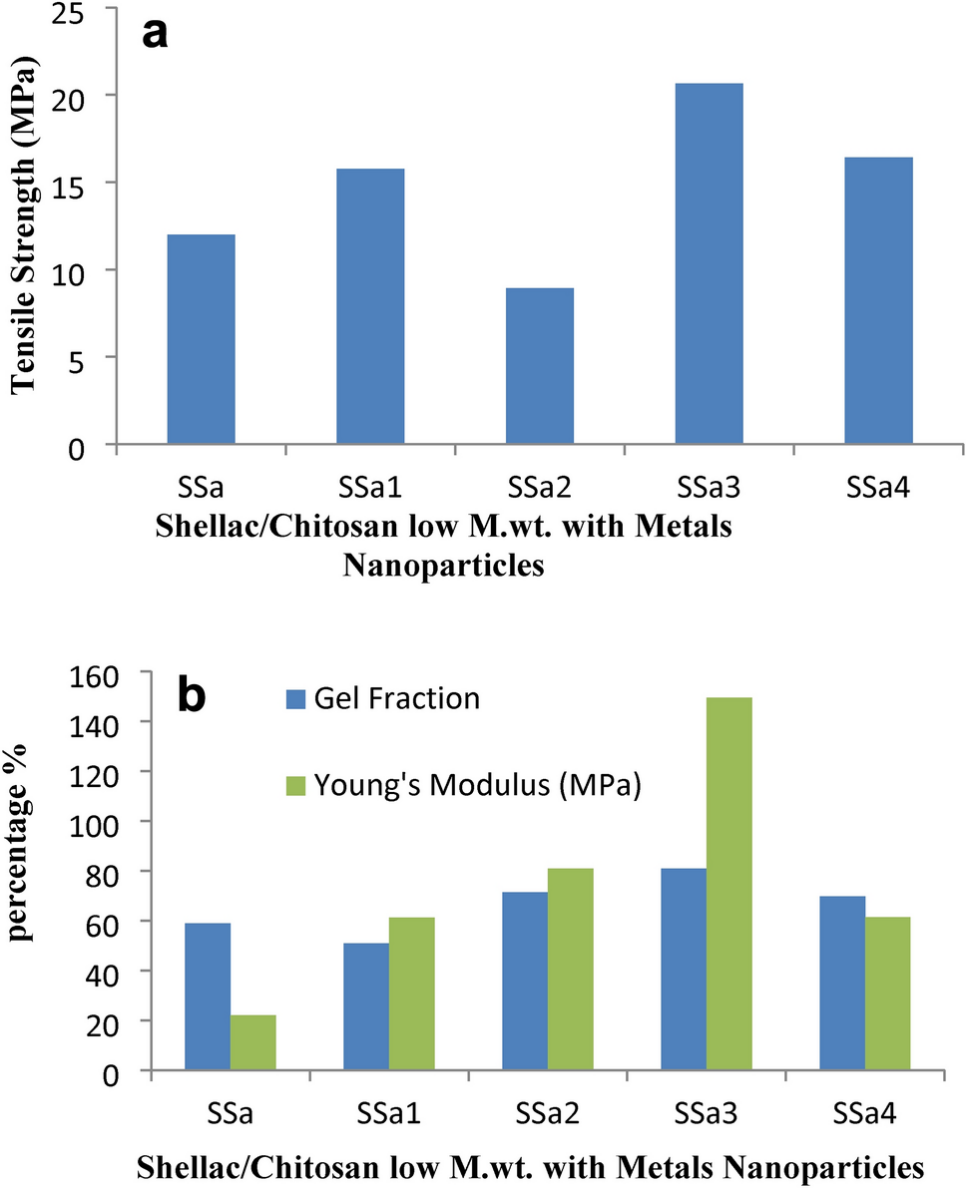
(**a**) Tensile strength Shellac/Chitosan low M.wt. films with metals nanoparticles. (**b**) Gel Fraction and Young Modulus of Shellac/Chitosan low M.wt. films with metals nanoparticles.

**Fig. 6:**
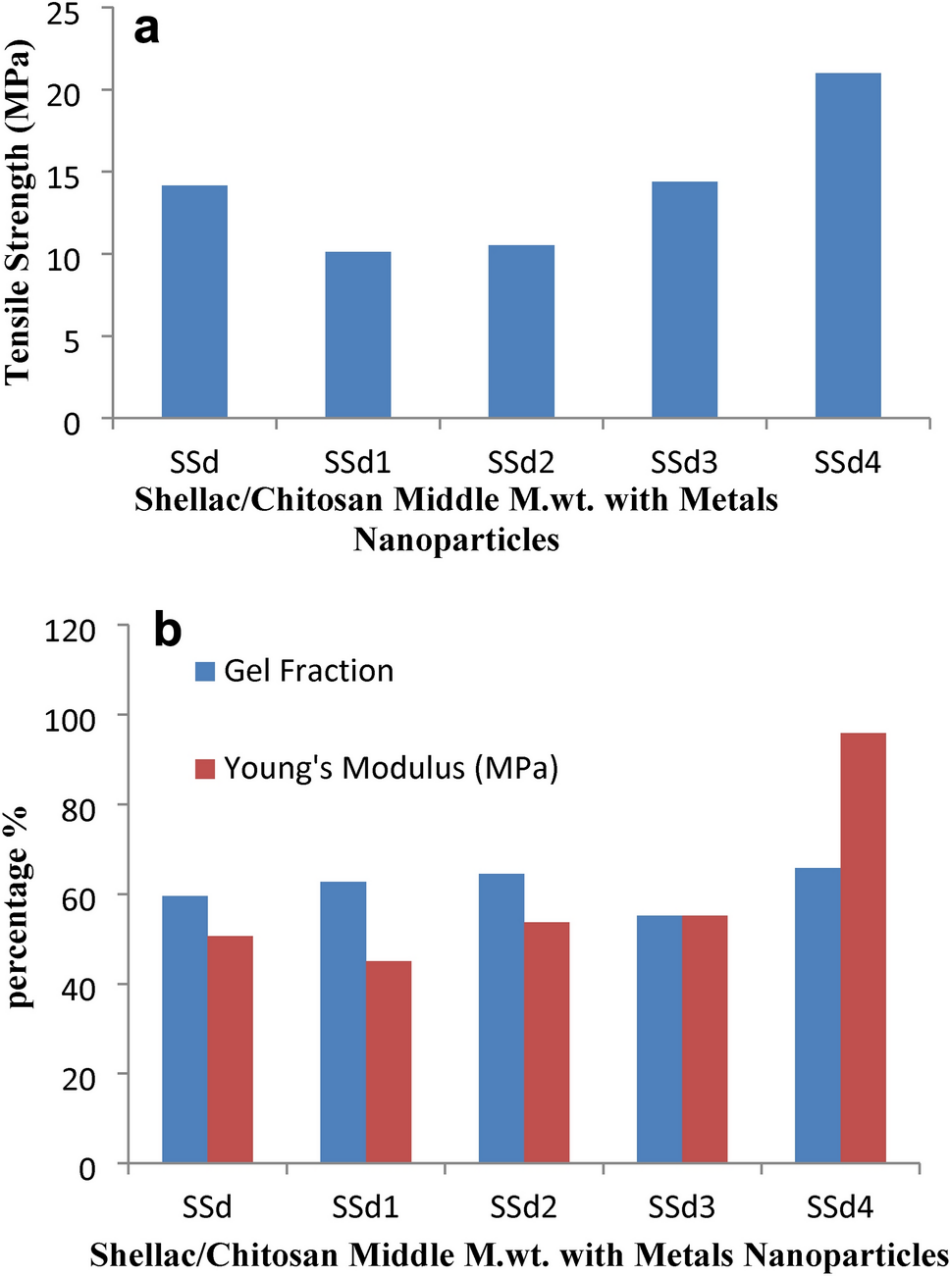
(**a**) Tensile strength Shellac/Chitosan Middle M.wt. films with metals nanoparticles. (**b**) Gel Fraction and Young Modulus of Shellac/Chitosan Middle M.wt. films with metals nanoparticles.

Chitosan/shellac composites incorporated with the spinel fillers improve Young’s modulus and some tensile strength values in comparison to unfilled chitosan. Tables 2, 3, and Figs. 5a,b, and 6a,b shows the mechanical properties of the chitosan/shellac-CuAl_2_O_4_ and ZnAl_2_O_4_ composite. Figure 5a,b shows that 0.05% of CuAl_2_O_4_ and ZnAl_2_O_4_ increased the TS and Young’s modulus compared to the chitosan/shellac film. The film properties improvement reflects the bonding between composite films. Additionally, the chitosan/shellac-CuAl_2_O_4_ composite has superior properties compared to the chitosan ZnAl_2_O_4_ composite. In general, 0.05% spinel addition to chitosan/shellac provides a composite that has better qualities than 0.1% addition. Middle M.wt. chitosan provides a composite with better properties compared to that of low M.wt. (except for Young’s modulus), but the improvement in tensile strength and Young’s modulus were more pronounced in the case of using low M.wt. chitosan. Sample (0.05% CuAl_2_O_4_ composite with low M.wt. chitosan/shellac, SSa3) shows the most favorable properties, compared to the other composite samples, with a 72% increase in tensile strength; and 581% increase in Young’s modulus; with a comparable air permeability as shown in Fig. 5a,b. The inclusion of the CuAl_2_O_4_ and ZnAl_2_O_4_ was implied to have the potential to enhance the films’ mechanical characteristics. Our outcomes were comparable to those shared with another work in which the TS of various nanocomposite films using an organoclay combination Fasihnia, et al.^5^ and Tohamy, et al.^55^. Sample (0.1% CuAl_2_O_4_ composite with middle M.wt. chitosan/shellac, SSd4) shows the most favorable properties, compared to the other composite samples, with a 48% increase in tensile strength; 89% increase in Young’s modulus as shown in Fig. 6a,b; with a comparable air permeability; but there is minimal decrease in burst strength, Table 3. Also, the sample (0.1% ZnAl_2_O_4_ composite with middle M.wt. chitosan/shellac, SSd2) shows a 5.92% increase in Young’s modulus compared to middle M.wt. chitosan/shellac composite; with a comparable air permeability; but there are minimal decreases in tensile and burst strength. The addition of ZnAl_2_O_4_ or CuAl_2_O_4_ more than 0.1% to chitosan/shellac composite film gave bad films^40^. It was suggested that the CuAl_2_O_4_ and ZnAl_2_O_4_ addition may improve the mechanical properties of the films. This improvement in the film may be due to the interaction between the CS/SH matrix and CuAl_2_O_4_ and ZnAl_2_O_4_. This could enable CuAl_2_O_4_ and ZnAl_2_O_4_ to be infiltrated into the membrane network, causing a cross-linked effect by forming a coordinated bond between Cu or Zn with OH NH C=O of chitosan/shellac matrix. Meanwhile, the molecular forces between polymer chains were reduced and the tensile strength and gel fraction of the films were increased^75^.

**Table 2:** The mechanical properties of chitosan (low M.wt.)/shellac/ZnAl_2_O_4_ and CuAl_2_O_4_ composite films.

Designation of film formulation	Reaction conditions				Thick-ness	Swel-ling (%)	Percentage strain at maximum load	Burst Strength (kg/cm^2^)	Air permea-bility (s)	Biodeg-radation %
	SH 5%	CH LM 2%	Zn/C (g)	Cu/C (g)						
SSa	10	90	00	00	0.25 ± 0.03	13.95 ± 1.39	21.93 ± 0.05	2.65 ± 0.31	Nil	57.09 ± 0.52
SSa1	10	90	0.05	00	0.23 ± 0.01	3.72 ± 1.22	61.23 ± 2.25	2.25 ± 0.04	Nil	45.31 ± 0.33
SSa2	10	90	0.1	00	0.25 ± 0.01	10.56 ± 1.15	80.88 ± 3.82	1.65 ± 0.12	Nil	38.08 ± 0.34
SSa3	10	90	00	0.05	0.26 ± 0.01	50.14 ± 2.15	149.36 ± 2.86	2.25 ± 0.05	Nil	41.11 ± 0.16
SSa4	10	90	00	0.1	0.24 ± 0.01	15.67 ± 1.54	61.27 ± 1.41	2.1 ± 0.11	Nil	42.16 ± 1.05

*SH* Shellac, *CH* Chitosan, *LM* Low M.wt.

**Table 3:** The mechanical properties of chitosan (middle M.wt.)/shellac/ZnAl_2_O_4_ and CuAl_2_O_4_ composite films.

Designation of film formulation	Reaction conditions				Thick-ness	Swel-ling (%)	Percentage strain at maximum load	Burst strength (kg/cm^2^)	Air permea-bility (s)	Biodeg-radation %
	SH 5%	CH MM 2%	Zn/C (g)	Cu/C (g)						
SSd	10	90	00	00	0.24 ± 0.01	15.63 ± 2.31	29.40 ± 4.66	4.15 ± 0.41	Nil	39.17 ± 0.21
SSd1	10	90	0.05	00	0.26 ± 0.02	7.48 ± 1.35	16.88 ± 1.49	1.75 ± 0.22	Nil	37.50 ± 0.14
SSd2	10	90	0.1	00	0.27 ± 0.01	11.40 ± 0.74	13.24 ± 0.81	2.00 ± 0.06	Nil	39.37 ± 0.0.31
SSd3	10	90	00	0.05	0.26 ± 0.01	10.29 ± 0.38	29.29 ± 1.13	2.75 ± 0.16	Nil	39.66 ± 0.61
SSd4	10	90	00	0.1	0.27 ± 0.01	10.80 ± 0.54	25.48 ± 3.20	3.3 ± 0.23	Nil	36.62 ± 0.22

*SH* Shellac, *CH* Chitosan, *MM* Middle M.wt.

#### Swelling and gel fraction analysis

Tables 1, 2 and 3 show how the percentage swelling and gel fraction of the CS/shellac and CS/shellac spinel films are affected by the CS/shellac and CS/shellac spinel weight ratio. Table 1 clearly show that adding 10% shellac to CS (low M.Wt.) (V/V) increases the films’ percent swelling and gel fraction, which rise from 7.03 and 50.61 to 13.95 and 58.95%, respectively. Furthermore, the percentage of swelling and gel fraction of the films increased from 6.52 and 51.68 to 15.63 and 59.58%, respectively, upon adding 10% shellac to CS (middle M.Wt.).

It appears that the chitosan macromolecules and the shellac macromolecules combine to form a secondary bond. So that chitosan can form H-bonding networks and stronger molecular chain entanglements inside the structure of the film. The swelling characteristics and gel fraction extents decreased to 2.52 and 53.68% when the shellac ratio was raised from 10 to 20%. This indicates a slow dissociation of the film structure as a result of a reduction in the entanglements between the chitosan chains and, as a result, a reduction in the intermolecular bonds between these chains.

Moreover, Table 2 shows the effect of adding 0.05 and 0.1% ZnAl_2_O_4_ or CuAl_2_O_4_ shellac/CS (low M.Wt.) composites (W/V) films. Adding ZnAl_2_O_4_ or CuAl_2_O_4_ shellac/CS (low M.Wt.) reduces swelling from 13.95 to 10.56% and increases the gel fraction of the films from 58.95 to 71.40% when 0.1% ZnAl_2_O_4_ is added. However, adding 0.05% CuAl_2_O_4_ increases both swelling and gel fraction of the films from 13.95 and 58.95 to 50.14 and 80.18%, respectively. The increase in gel fraction evaluates the increase in polymer percentage (CS and SH) involved in the covalent and physical cross-linking reaction by covalent bonds.

Table 3 and Fig. 6a,b shows the effect of addition 0.05 and 0.1% ZnAl_2_O_4_ or CuAl_2_O_4_ to shellac/CS (middle M.Wt.) composites (W/V) films on the mechanical properties. When ZnAl_2_O_4_ and CuAl_2_O_4_ are added to shellac/CS (middle M.Wt.) the percentage of swelling decreased from 15.63 to 11.40% and the gel fraction of the film increased from 59.58 to 64.40% when 0.1% ZnAl_2_O_4_ is added. Similarly, when 0.05% CuAl_2_O_4_ is added, the swelling percentage decreases from 15.63 to 10.80% and the gel fraction increases from 59.58 to 65.82%. Additionally, ZnAl_2_O_4_ or CuAl_2_O_4_ combined with shellac/CS (low or middle M.Wt.) film structure at concentrations of 0.05 and 0.1% leads to an increase in the film gel fraction or firmness, and a slight decrease in the degree of swelling in that film when compared to film formulations that only contain shellac as a bio-additive to chitosan. The results of the effect on metal NPs agree with a previous study^40^. This suggests that ZnAl_2_O_4_ or CuAl_2_O_4_ and shellac in the CS film structure have a synergistic effect.

#### Scanning Electron Microscopy (SEM)

**Fig. 7:**
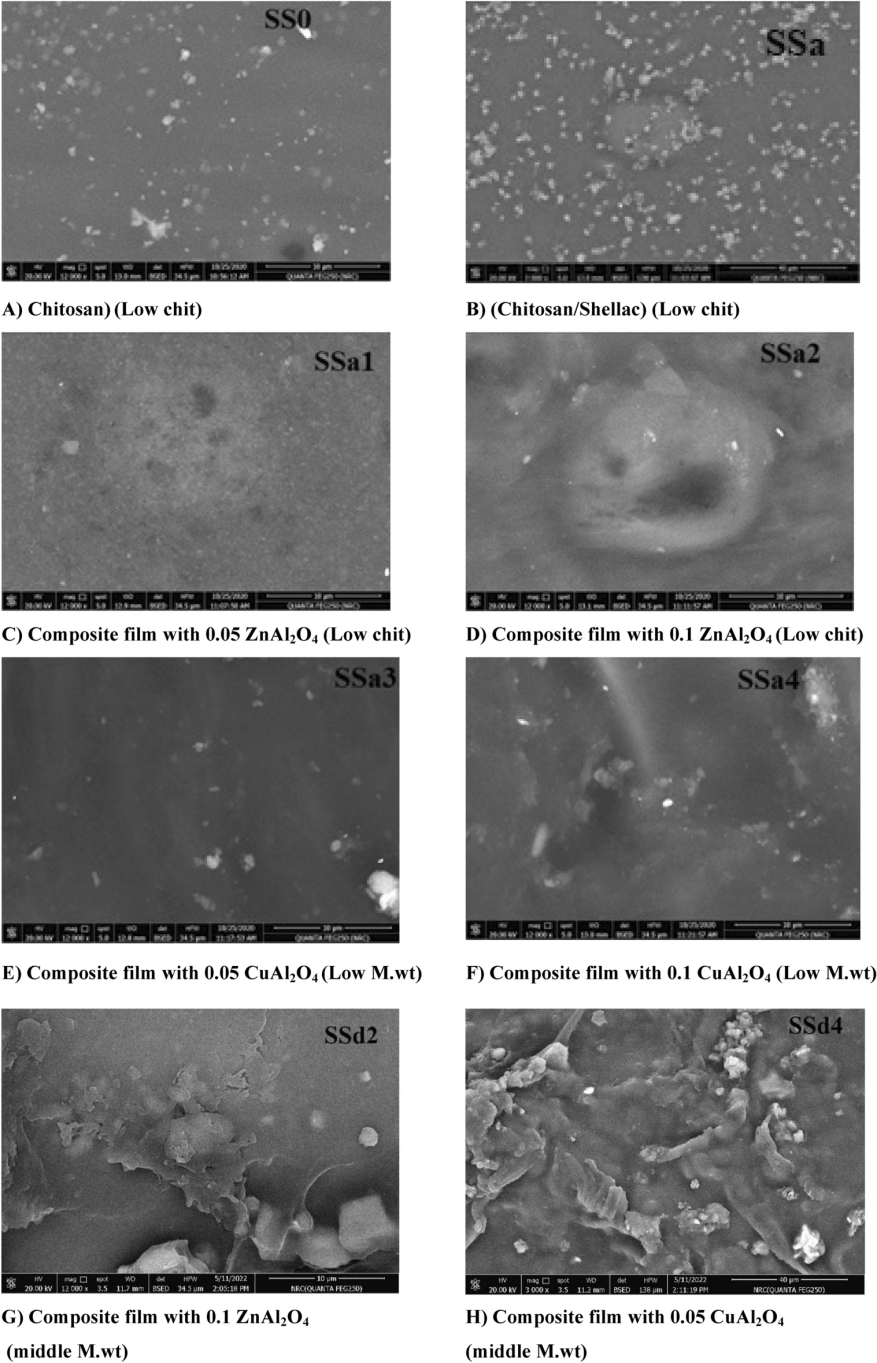
SEM of the CS, CS/shellac, and CS/shellac spinel composite showed that aggregation of ZnAl_2_O_4_ or CuAl_2_O_4_ with chitosan/shellac composites formed macromolecules.

SEM was used to identify the morphology of the different obtained samples. SEM images of CS and CS/shellac in Fig. 7A,B reveal that both films do not show any differences in morphology. The two films show the formation of the microspheres and agglomerated nanoparticles with a rough distribution^76^. Figure 7C,D for 0.05 and 0.1% ZnAl_2_O_4_ composites; and Fig. 7E,F for 0.05 and 0.1% CuAl_2_O_4_ composites, samples SSa(1–4) respectively, show the formation of homogeneous films with good distribution of the spinel aluminate nanoparticles. The non-porous structure was exposed so, enhanced water resistance and mechanical properties were assumed.

Figure 7G,H for samples no. SSd2 and SSd4 (0.1% ZnAl_2_O_4_ and CuAl_2_O_4_ composites with middle M.wt. CS), respectively, show that the morphology displays the interlaced assembly of the attained films. It can be observed that the samples were composed of aggregated nanoparticles with rough sphere surfaces and internal porosity, but free of cracks. This could explain the superior improvement in mechanical properties for low M.wt. chitosan spinel composite than the middle M.wt. chitosan.

As a result of SEM pictures, we can notice that the ZnAl_2_O_4_ and CuAl_2_O_4_ spinel nanoparticles are as well as the strong binding of the bio-additives molecules to the CS/ shellac blend polymeric structure which means that macromolecules are formed by formation of the complexes between chitosan/shellac with ZnAl_2_O_4_, and CuAl_2_O_4_ spinel nanoparticles. SEM of the CS, CS/shellac and CS/shellac spinel composite showed aggregation of ZnAl_2_O_4_/CuAl_2_O_4_ spinel by formation complexes with chitosan/shellac which form the biological macromolecules^40^. The more homogeneous surface appearance of the pure chitosan and CS/shellac films suggested that the biopolymer matrix had a crystalline structure. On the other hand, NPs of ZnAl_2_O_4_ and CuAl_2_O_4_ spinel nanoparticles accumulation in the biopolymer matrix is probably the reason why the shape of nanocomposite films was diverse when compared to the pure chitosan and CS/shellac films film^40^.

### FTIR Spectroscopy

**Fig. 8:**
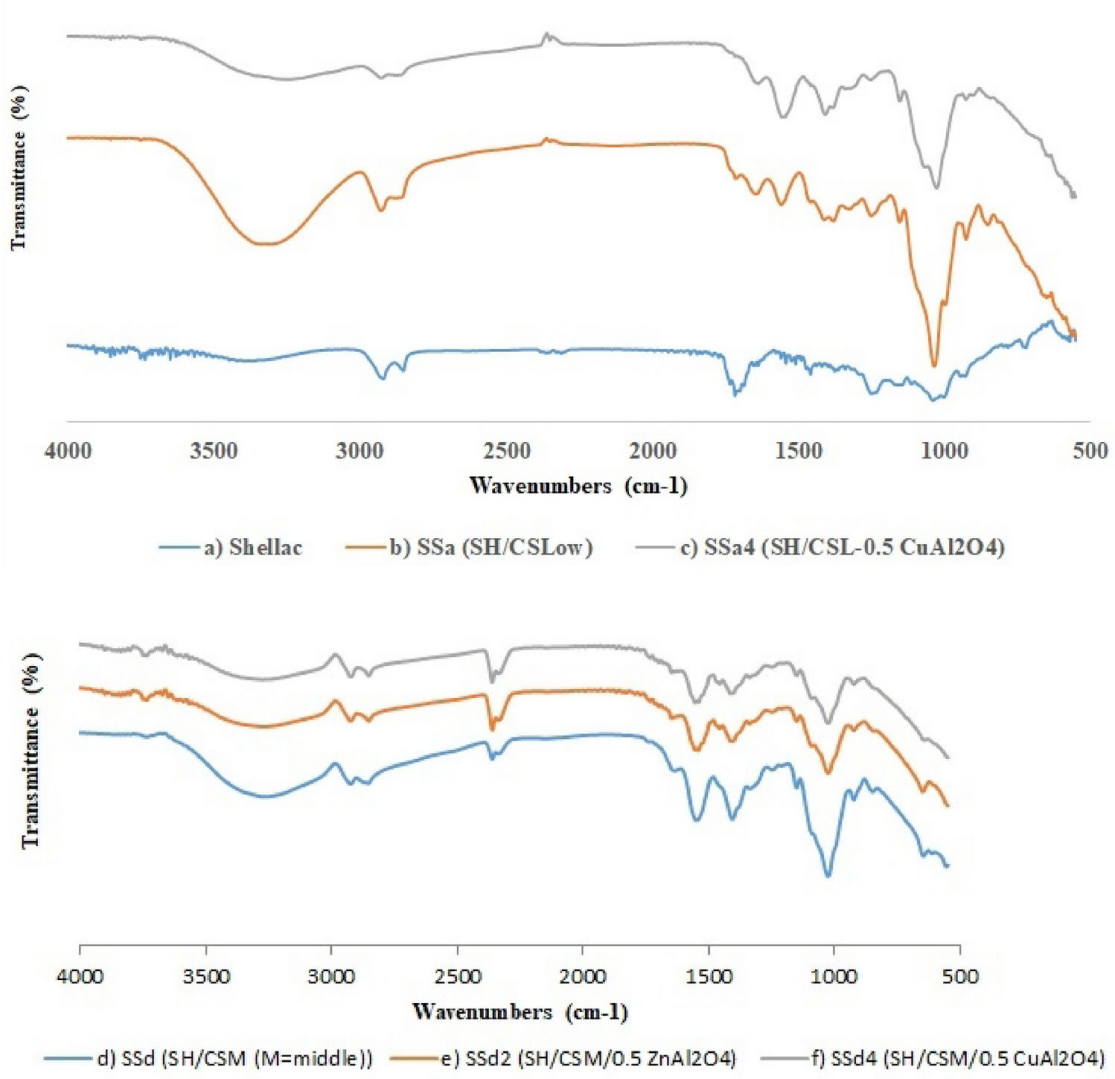
FTIR of (**a**) shellac (SH), (**b**) the chitosan low (CSL)/SH, (**c**) CSL/SH with spinel composite, (**d**) chitosan middle (CSM)/SH and (**e**) and (**f**) CSM/SH with 0.1% spinel composite ZnAl_2_O_4_ or CuAl_2_O_4_ respectively.

The FTIR spectrum of shellac, Fig. 8a, exhibited the characteristic bands around 2859, 1715, and 1056 cm^−1^ assigned to the stretching methylene, stretching carbonyl, and the absorption peak of the ether bond (C–O) respectively. This was explained by the shellac structure’s abundance of ethylene and carbonyl groups^56^.

Figure 8b for shellac/CS (low M.Wt.) composite exhibited the typical reported characteristic peaks for chitosan. The bands around 3200 and 3500 cm^−1^ are attributed to the overlapping of –OH and –NH symmetric stretching vibrations, and duplet bands at ~ 2900 cm^−1^ are ascribed to chitosan’s symmetric and asymmetric C–H stretching. The C=O stretching and N–H bending vibrations of chitosan are represented by the two peaks at 1662 and 1551 cm^−1^, respectively^73,76^.

The sharp absorption peak at 1061 cm^−1^ corresponded to the overlapping of C–O ether bond vibration for both chitosan and shellac. In comparison with the FT-IR bands of pure shellac, observations reveal that the peak intensities at 2859 and 1715 cm^−1^ were decreased in the composite, which implied the effective combination of shellac within CS film. The FT-IR spectra of the shellac/CS (low and middle M.Wt.) film (Fig. 8a–f) showed variation in the intensity of FTIR peaks. Figure 8b,d revealed increasing in the broad band around 3200 and 3500 cm^−1^ than Fig. 8a; these are attributed to the increase of –OH and –NH groups, resulting in the reaction between chitosan and shellac. In addition, the electrostatic interaction occurred between the positive charge of CS and the negative charge of shellac without the forming new chemical bonds between them^77^.

When comparing the CS/SH film to the CS/SH/ZnAl_2_O_4_ or CuAl_2_O_4_ film, the ZnAl_2_O_4_ or CuAl_2_O_4_ nanoparticles are embedded into the CS/SH matrix as evidenced by the considerably in Fig. 8c,e,f revealed decreasing in the broad bands around 3200 and 3500 cm^−1^ and the higher C–O–C peaks at 1000 cm^−1^ with decreasing the intensity and shifted of –COO^−^ peak at 1662 cm^−1^. Consequently, the films containing the fillers had strong hydrogen and ionic bonding^76^, which may be attributed to the reaction between ZnAl_2_O_4_ and CuAl_2_O_4_ with –OH, –NH, and –C=O groups results in formation of coordinated bonds between the transition metal, Zn or Cu (the coordination centers) and the lone pair of electrons of OH, NH and C=O groups of chitosan/shellac (as ligands or complexing agents)^78^, to form a coordination complex (metal complex)^79^. These results confirm and reinforce the results of mechanical properties such as tensile strength, Young’s Modulus, bursting strength, etc. gel fraction, SEM, and TGA/DTG curves in which the melting point shifted into a high melting point in the DTG curve.

Figure 8c,d shows the FT-IR spectra of shellac/CS with ZnAl_2_O_4_ and CuAl_2_O_4_. The FTIR spectrum exhibits the characteristic peaks of shellac/CS (Fig. 8b) at around 3400, 2900, 1650, and 1060 cm^−1^. Metal-O stretching frequencies in the range of 500–900 cm^−1^ were recognized for M–O, Al–O, and M–O–Al (where M is Zn or Cu) for ZnAl_2_O_4_ and CuAl_2_O_4_ composites. The observed variation in band intensities between shellac/CS and that one with ZnAl_2_O_4_ (Fig. 8e) or CuAl_2_O_4_ (Fig. 8c,f) suggests that the Zn or Cu and Al ions interact with the polymer functional groups during composite formation. The strong interaction of Zn or Cu-Al_2_O_4_ with the amine groups in chitosan increases the intensity of the band at 1365 and 1515 cm^−1^ in the spectrum, which is attributed to the N–O stretching vibration.

#### The UV–visible spectra

**Fig. 9:**
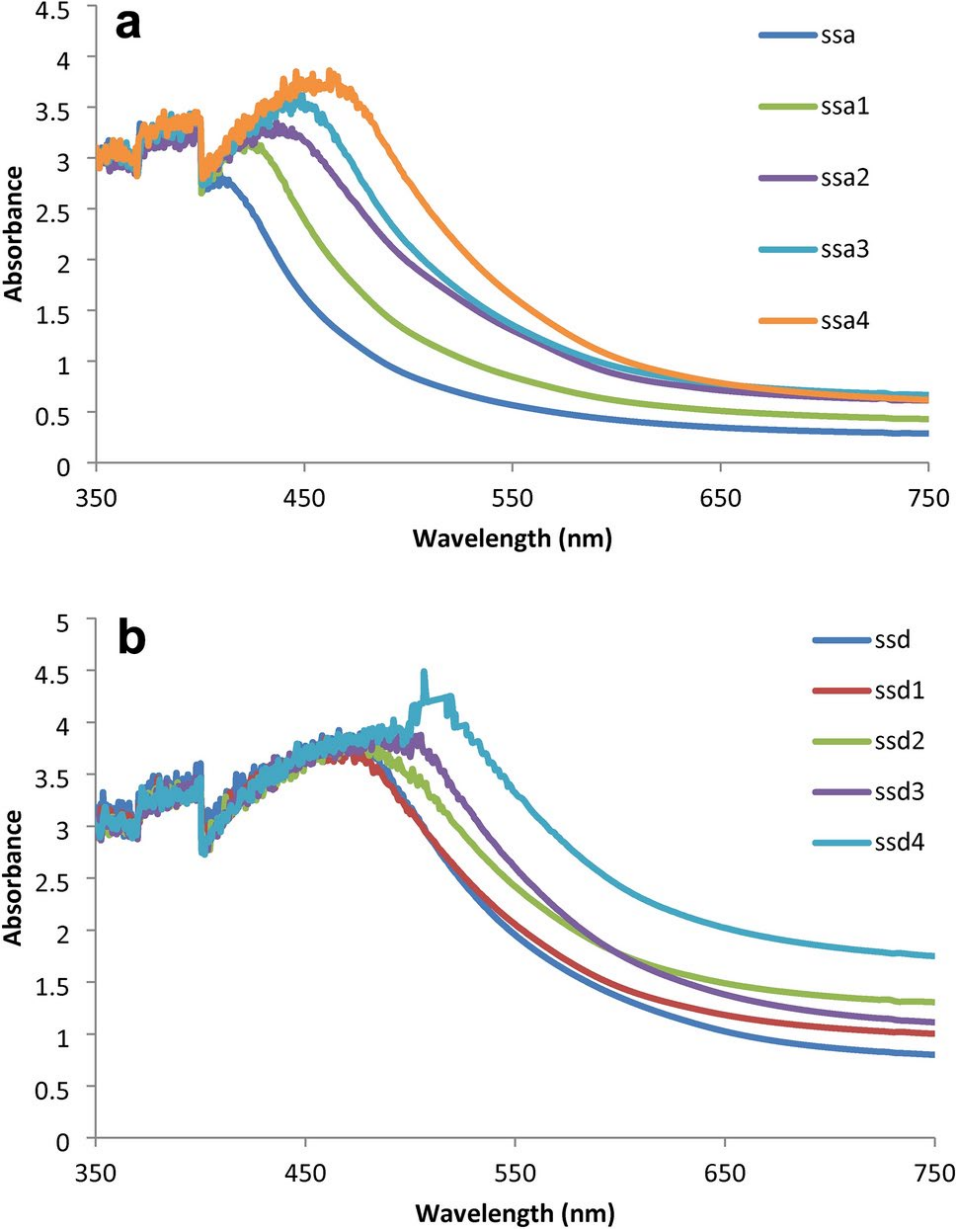
(**a**) Ultraviolet and visible spectrum for compounds SSa (1–4). (**b**) Ultraviolet and visible spectrum for compound SSd (1–4).

The shellac/chitosan UV–visible spectra, as well as their modification with Zn and Cu spinel nanoparticles to form complexes, are shown in Fig. 9a,b. The UV and visible light transmittances of the CS/SH-ZnAl_2_O_4_ or CuAl_2_O_4_ composite films were lower than those of the pure CS/SH film (Fig. 9a,b), indicating that the addition of ZnAl_2_O_4_ or CuAl_2_O_4_ and SH particles can significantly increase the films’ resistance to UV and visible light. The spectra of CS/SH-ZnAl_2_O_4_ or CuAl_2_O_4_-complexes show high light absorption at a wavelength of 400–600 nm, which demonstrated a low transmittance in the region of visible light with shifting to a higher wavelength more than chitosan/shellac upon the introduction of the Zn and Cu NPs to the composites, which can be attributed to CT (charge transfer) from the ligand to the metal^76^. Furthermore, as the ZnAl_2_O_4_ or CuAl_2_O_4_ content got higher, the visible light transmittance steadily reduced. Because of its multi-layered sheet structure, ZnAl_2_O_4_ or CuAl_2_O_4_ produces strong intermolecular forces that help the ZnAl_2_O_4_ or CuAl_2_O_4_ particles disperse in the CS/SH matrix and increase the composite film’s roughness, both of which reduce light transmission^76^, confirming that the transmittance of composite films can be effectively decreased by the addition of ZnAl_2_O_4_ or CuAl_2_O_4_ particles.

#### Thermogravimetric analysis (TGA/DTG)

**Fig. 10:**
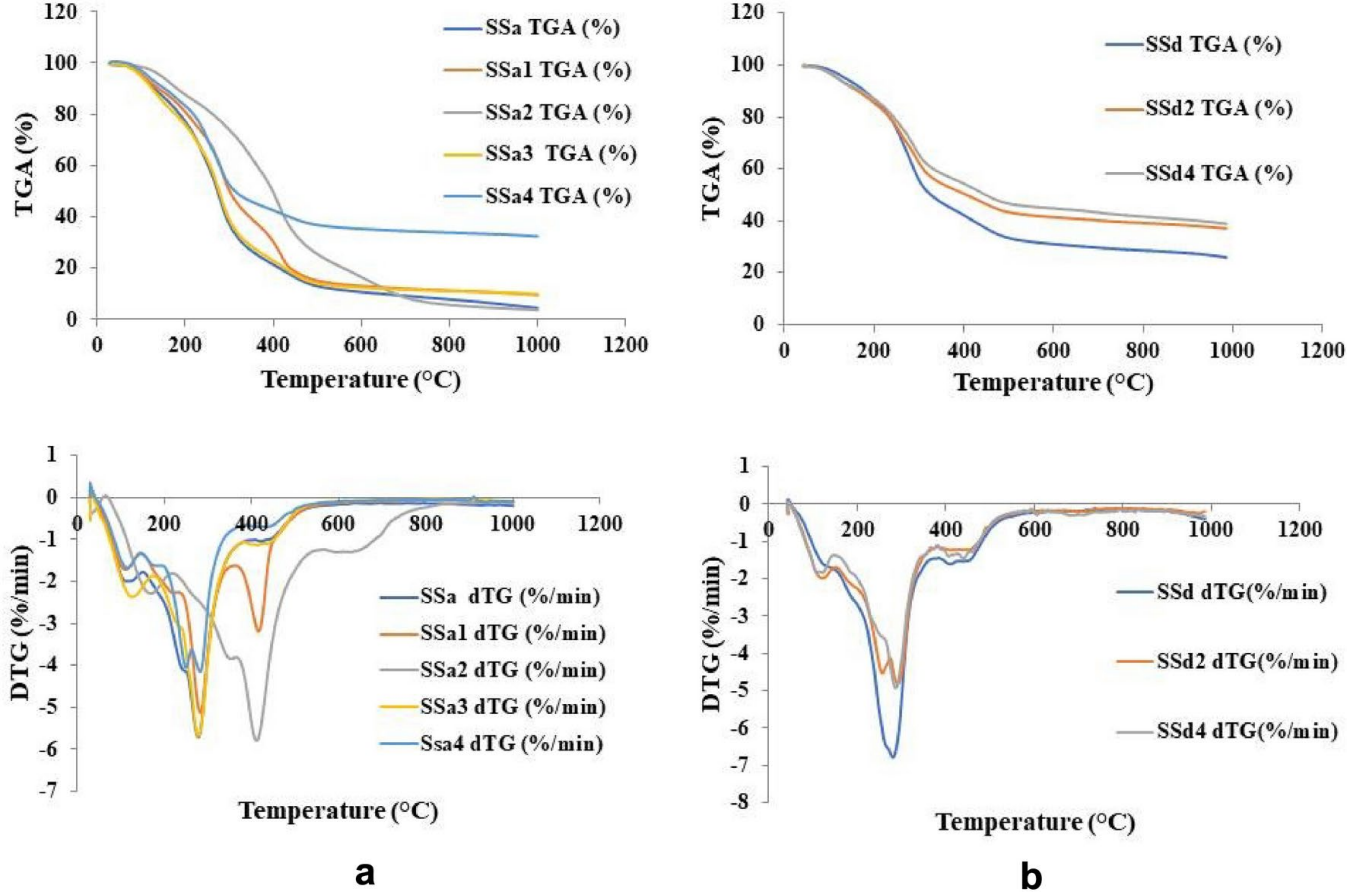
(**a**) TGA and DTG (%/min) of the CS (low M.Wt.), CS/shellac, and CS/shellac spinel composite. (**b**) TGA and DTG (%/min) of the CS (middle M.Wt.), CS/shellac, and CS/shellac spinel composite.

TGA and DTG curves for shellac/CS (low M.wt.) composite with 0.05 or 0.1% ZnAl_2_O_4_ or CuAl_2_O_4_ composites, samples SSa (1–4), were depicted in Fig. 10a. TGA and DTG curves for shellac/CS (middle M.wt.) composite with 0.05 or 0.1% ZnAl_2_O_4_ or CuAl_2_O_4_ composites, samples SSd (1–4), were depicted in Fig. 10b.

Generally, TGA thermograms for the composite samples displayed three main stages of mass loss in the same temperature range during the thermal decomposition process, and all the composite samples display three main stages of differential thermogravimetry, or DTG, it is a TGA derivative that calculates the rate at which weight varies with temperature. It is useful for determining the temperature at which a substance undergoes a certain thermal event, such as combustion or breakdown Table 4.

**Table 4:** Differential thermogravimetric analysis (DTG) of the prepared samples.

Code	DTG peak, °C	Stage	Code	DTG peak, °C	Stage
SSa	91.91	1st	SSd	112.98	1st
	269.04	2nd		269.01	2nd
	400.96	3rd		405.80	3rd
SSa1	93.18	1st	SSd2	104.50	1st
	275.35	2nd		251.9–283.1	2nd
	407.64	3rd		428.65	3rd
SSa2	149.49	1st	SSd	100.02	1st
	399.99	2nd	4	278.93	2nd
	624.60	3rd		420.07	3rd
SSa3	110.25	1st			
	271.19	2nd			
	406.58	3rd			
SSa4	101.68	1st			
	244.79–273.9	2nd			
	415.72	3rd			

As shown in Fig. 10a and Table 4, the initial 8–14% mass loss around 50–145 °C and differential thermogravimetric analysis (DTG) around 91–149 and 100–113 °C was observed for shellac/CS (low M.wt.) composite and shellac/CS (middle M.wt.) composite respectively, with maximum temperatures of 106 °C. This stage is attributed to the vaporization of physically and/or chemically adsorbed water on the composite surface. Also, in this stage, the dehydration of chitosan has occurred.

The second major weight-loss stage was between 191–391 °C with maximum temperatures of 202–277 °C and an average mass loss of 51–69%, and DTG in the area of 244–400 and 251–283 °C was recorded for shellac/CS (low M.wt.) composite and shellac/CS (middle M.wt.) composite respectively. This stage is ascribed to the decomposition, deacetylation, and depolymerization of the organic hydrocarbon backbone of the shellac and chitosan segment.

The degradation of different SH/CS composites occurred at the third stage from 370–610 with maximum temperatures 416–445 °C and an average mass loss of 12–20% and DTG in the area of 400–624 and 405–675 °C for shellac/CS (low M.wt.) composite and shellac/CS (middle M.wt.) composite respectively, is attributed to the pyrolytic degradation of the polymeric network and release of organic matter in the carbonization process^72^.

The incorporation of ZnAl_2_O_4_ or CuAl_2_O_4_ enhanced the thermal stability of the SH/CS composite; as a result, the remaining mass of the composite was increased. Figure 10a demonstrates that ZnAl_2_O_4_ provides superior thermal stability than CuAl_2_O_4_. At 0.05 and 0.1% aluminate addition; Zn provides higher thermal stability for composite than Cu till 450 and 680 after that they have comparable stabilization.

As a conclusion, TGA for chitosan (CS middle M.wt.) composites, Fig. 10b, shows comparable curves for that of low M.wt. CS with the same degradation stage and the same thermal decomposition process. Middle M.wt. CS provides a more stable composite than that for low M.wt. CS. The residual weight was about 90–93%, 52–62%, and 30–45% at 200, 400, and 800 °C, respectively, compared to 76–87%, 22–50%, and 8–12% for low M.wt. CS at the same temperature range. ZnAl_2_O_4_ and CuAl_2_O_4_ composites afford higher thermal stability than shellac/CS composite at temperatures higher than 300 °C, with slightly superior properties for CuAl_2_O_4_ than ZnAl_2_O_4_.

From the DTG curves; the DTG peaks shifted into a high wavelength in DTG curves after treatment of CH/SH with CuAl_2_O_4_ and ZnAl_2_O_4_-NPs which confirmed the chemical reaction that was referred to them in the mechanical analysis, SEM, and FTIR to form coordination bonds between CS/SH and CuAl_2_O_4_ or ZnAl_2_O_4_^12,41,46,49,78,79^. So, these results confirm the success of the methods of the mixing used in this study as in the suggested mechanism Fig. 16. Additionally, it is evident from differential TGA (DTG) curves, which show the link between temperature and mass loss rate, that all thermochromic powder mass loss rates clearly rise beyond 250 °C. The aforementioned experiment demonstrates that these composites primary pyrolysis processes take place above 250 °C, demonstrating their comparatively superior high-temperature stabilities. This is because the composites’ shell has strong thermal resistance, shielding the entire structure from damaging heat effects.

#### Biodegradation studies

A helpful and crucial method for determining if the prepared materials are compatible with the environment is the biodegradability test. For one week, a soil burial degradation method was used to assign the biodegradation of the composite films of chitosan and its treatments (Tables 1, 2 and 3). After one week of being buried in the soil, the chitosan sample demonstrated the exceptional ability to tower breakdown in soil with weight losses of 52.90 and 49.12% for chitosan low M.wt and Middle M.wt, respectively. This outcome is consistent with other earlier research^80^.

#### The images of fabricated samples

**Fig. 11:**
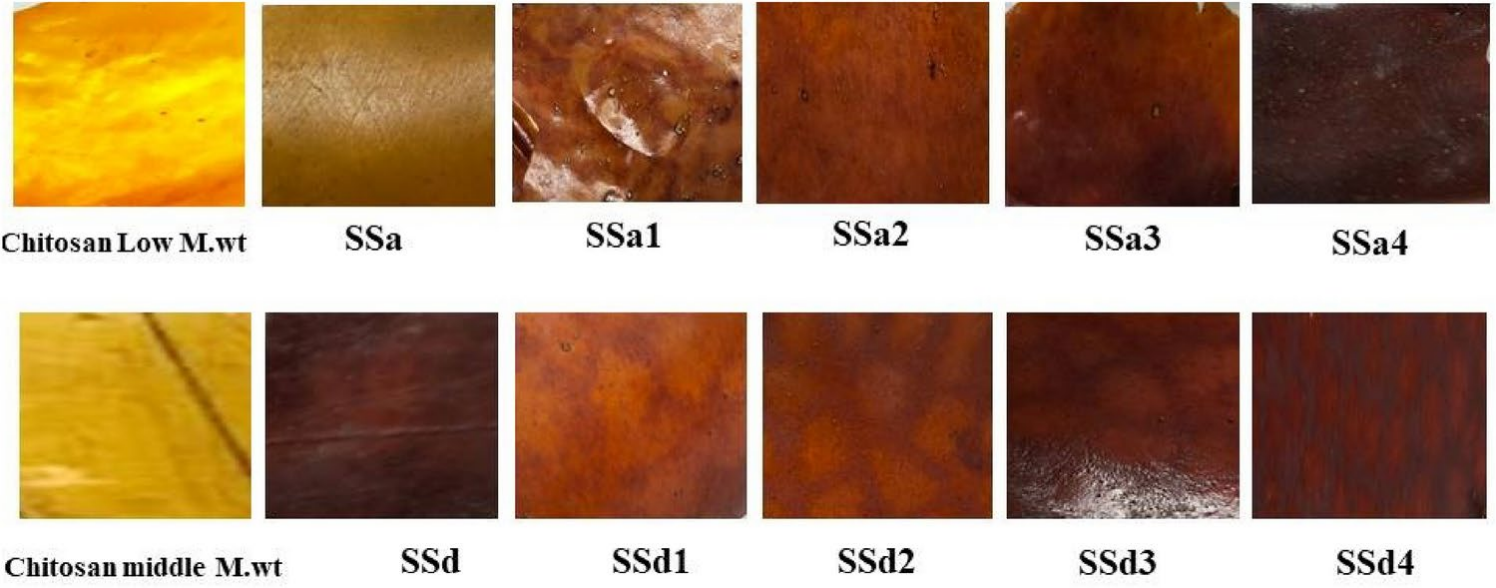
The images of fabricated samples of the CS (Low or Middle M.Wt.), CS/shellac, and CS/SH spinel composite.

The images of fabricated samples of the CS (Low or Middle M.Wt.), CS/shellac, and CS/SH spinel composite are shown in Fig. 11. The Photos of the produced composite films revealed a noticeable difference in the color which reflects the successful treatments and mixing between the CH and SH without/with ZnAl_2_O_4_ or CuAl_2_O_4_, Fig. 11.

#### Antimicrobial activity determination using the Agar diffusion technique

**Fig. 12:**
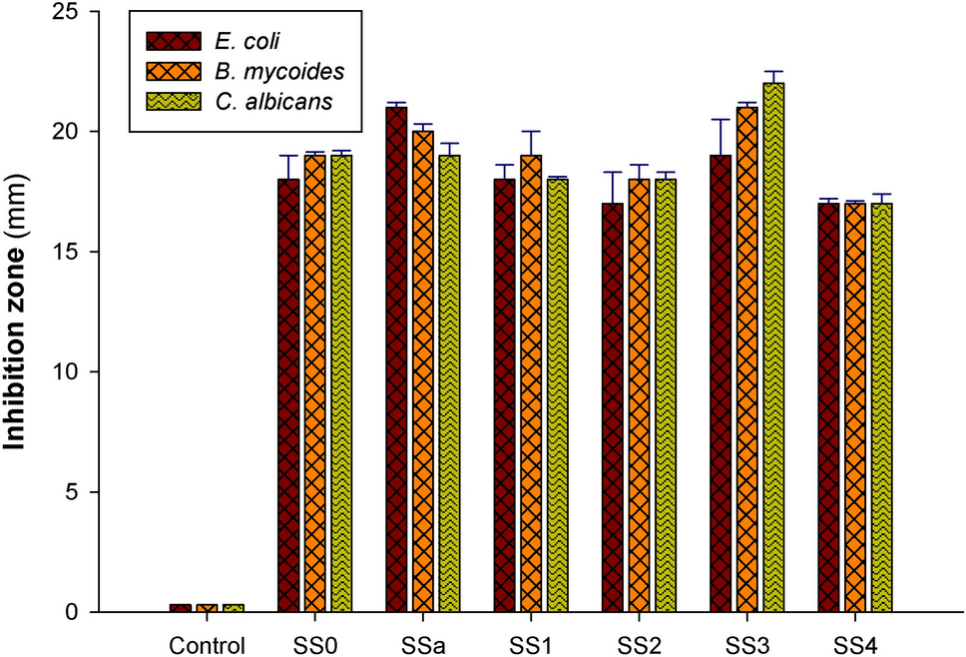
Testing the ability of produced composites to act as antibacterial agents using agar disc diffusion experiment.

**Fig. 13:**
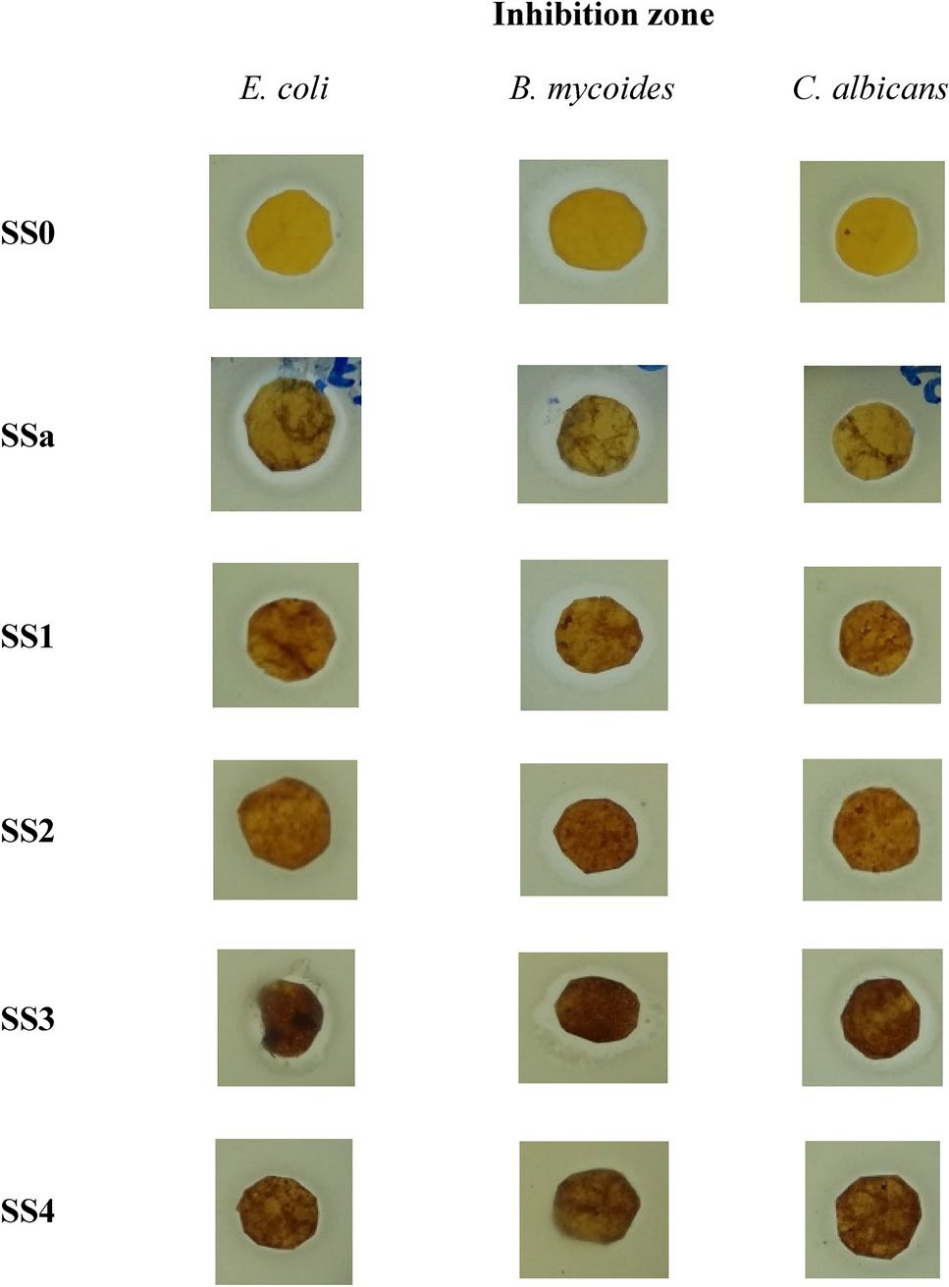
Photos of produced composites to act as antibacterial agents using agar disc diffusion experiment show the growth inhibition zones.

The antibacterial properties of shellac, chitosan, and spinel (ZnAl_2_O_4_ or CuAl_2_O_4_) mixed sheets were examined in this work against *E. coli* and *B. mycoides* as Gram-negative and Gram-positive bacteria, respectively, and against the non-filamentous fungus *C. albicans*. By measuring the growth inhibition zone around the discs on the sheets, the antibacterial activity was determined in millimeters. Table 5 and Figs. 12 and 13 display the results, demonstrating the antibacterial efficacy of the different composites that were created. The results demonstrate that the concentration of various composite components affects the proportional effects of different treatments and percentages on the efficiency of microbial growth. As a function of inhibition zone diameter, composites of SSa (1:9 of shellac (5%) and chitosan (2%)) had the highest Gram-negative antibacterial activity against *E. coli* (21 mm), whereas composites of SSa3 (1:9 of shellac (5%) and chitosan (2%), and 0.05% of CuAl_2_O_4_) exerted the highest Gram-positive antibacterial activity against *B. mycoides* (21 mm), and *C. albicans* (22 mm). On the other hand, composites of SSa4 (1:9 of shellac (5%), chitosan (2%), and 0.1% of CuAl_2_O_4_), had the lowest antimicrobial activity (17 mm) against all the tested microbial species. Additionally, composites of SSa2 (1:9 of shellac (5%) and chitosan (2%), and 0.1% of the other spinel ZnAl_2_O_4_) showed the same attitude of SSa4 sheets with a little enhancement against *B. mycoides* and *C. albicans* (18 mm) (Table 5).

**Table 5:** Testing the ability of produced composites to act as antibacterial agents using agar disc diffusion experiment.

Sample	Components of sheet (%)				Inhibition zone (mm)		
	Shellac (5%)	Chitosan2% (Low Mwt.)	Spinel (%)		*E. coli*	*B. mycoides*	*C. albicans*
			ZnAl_2_O_4_/C	CuAl_2_O_4_/C			
Control	100	–	–	–	0	0	0
SS0	–	100	–	–	18 ± 1.05	19 ± 0.15	19 ± 0.20
SSa	10	90	00	00	21 ± 0.25	20 ± 0.15	19 ± 0.30
SSa1	10	90	0.05	00	18 ± 0.65	19 ± 1.06	18 ± 0.18
SSa2	10	90	0.1	00	17 ± 1.33	18 ± 0.55	18 ± 0.37
SSa3	10	90	00	0.05	19 ± 1.52	21 ± 0.26	22 ± 0.56
SSa4	10	90	00	0.1	17 ± 0.23	17 ± 0.11	17 ± 0.40

**Fig. 14:**
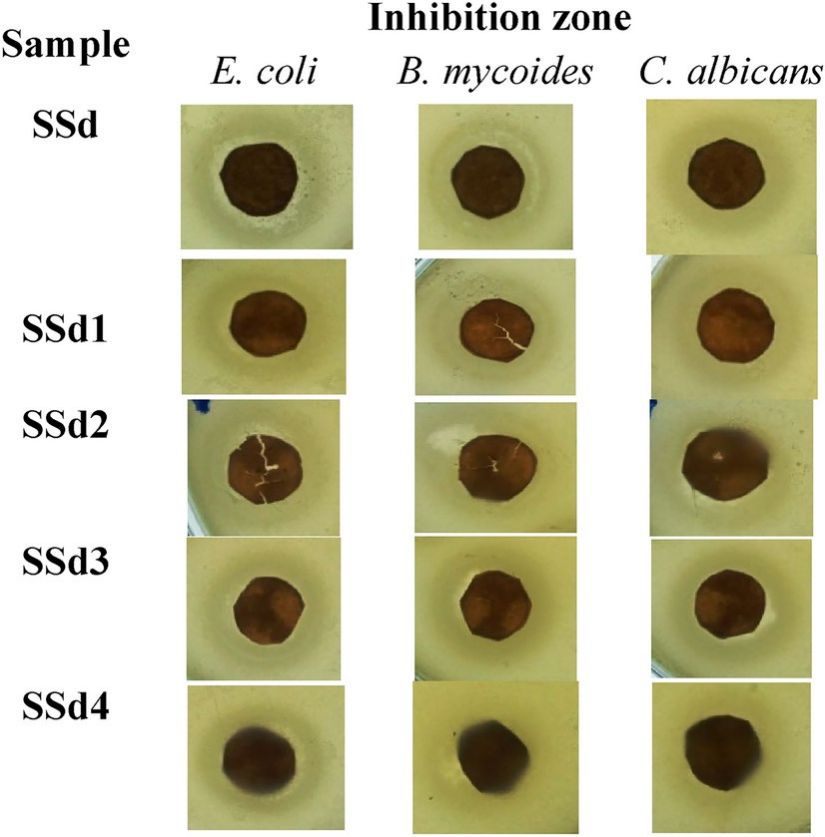
Photos of the growth inhibition zones on the antibacterial sheets made using the agar disc diffusion experiment.

To solve the limitations of utilizing traditional antimicrobial test techniques, such as the guarantee of proper contact of inoculums with treated surfaces, the dynamic shaking flask technique was urbanized for regular quality control and screening testing^81^. Because absorbance measurements are used instead of the traditional plating approach, this variation of the shaking flask method is faster to execute. It provides an indicator of bacterial efficiency within hours, whereas traditional plating procedures require at least three days to produce data. The antimicrobial effectiveness of the prepared sheets was moreover tested via the shake flask method with nutrient broth. The optical density of the media was measured over time to track microbial growth. Table 6 shows the percentage of microbial decrease with the treatment by different prepared sheets in control to the non-treated broth medium. The results obtained show the potency of chitosan sheets as antimicrobial agents against *E. coli, B. mycoides,* and *C. albicans*. The results also revealed the superiority of sheets prepared by mixing shellac (5%), chitosan (2%), and 0.05% of CuAl_2_O_4_ (SSa3) to get microbial inhibition percentages between 93 and 97%, which confirms the results obtained by the agar diffusion method. Conversely, sheets prepared by mixing shellac (5%), chitosan (2%), and ZnAl_2_O_4_ (SSa1 and SSa2) show the lowest microbial inhibition percentages (Table 6). Figure 14 shows the photos of the growth inhibition zones on the antibacterial sheets made using the agar disc diffusion experiment.

**Table 6:** The potency of produced composites to act as antibacterial agents using shake flask assay.

Sample	Growth inhibition (%)					
	*E. coli*		*B. mycoides*		*C. albicans*	
	3 h	72 h	3 h	72 h	3 h	72 h
Control	0	0	0	0	0	0
SS0	91.74 ± 2.05	97.25 ± 2.38	95.64 ± 1.24	96.88 ± 2.35	94.94 ± 3.08	95.35 ± 0.06
SSa	89.13 ± 3.05	95.88 ± 0.00	94.70 ± 2.06	96.10 ± 1.08	93.15 ± 2.46	90.30 ± 1.24
SSa1	88.26 ± 0.00	70.00 ± 1.58	95.02 ± 2.35	38.99 ± 2.31	94.35 ± 1.55	68.48 ± 1.23
SSa2	87.83 ± 1.33	66.67 ± 3.07	91.90 ± 0.00	69.79 ± 1.46	92.86 ± 2.65	56.36 ± 2.08
SSa3	93.04 ± 2.09	97.06 ± 1.45	95.33 ± 1.75	96.69 ± 0.08	94.35 ± 0.08	94.95 ± 3.55
SSa4	90.87 ± 0.15	94.71 ± 2.08	94.08 ± 3.55	97.08 ± 1.44	90.18 ± 1.67	65.45 ± 0.02

Control : (microorganism only).

**Fig. 15:**
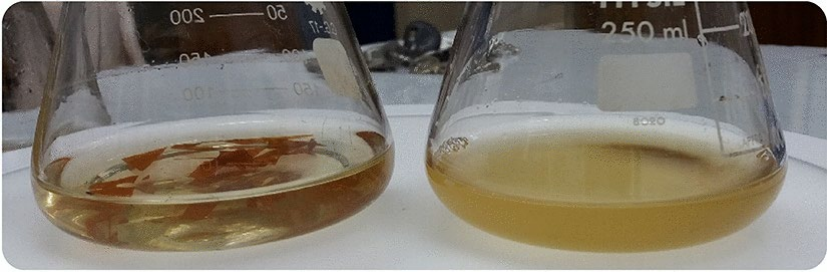
The shaking flask assay’s measurement of the growth density between treated sheets and control flasks.

This study looked also at the antibacterial activity of shellac, chitosan (middle molecular weight), and spinel (ZnAl_2_O_4_ or CuAl_2_O_4_) combined sheets against Gram-negative and Gram-positive bacteria, as well as *C. albicans*, a non-filamentous fungus. By measuring the growth inhibition zone surrounding the sheets’ discs, the antibacterial activity was estimated in millimeters. The antibacterial efficiency of various manufactured sheets was demonstrated in Table 7 and Fig. 15. The results reveal that different types of treatments and mixing percentages have varying proportionate effects on microbial growth efficiency, which depends on the concentration of different sheet components. Sheets of SSd showed the strongest Gram-negative antibacterial activity against *E. coli* (22 mm) as a function of inhibition zone diameter. Sheets SSd3 prevented *C. albicans* development as a non-filamentous fungus to a maximum of 20 mm. Additionally, against *B. mycoides* (16 mm) and *C. albicans*, sheets of SSd4 exhibited a similar attitude (Table 7).

**Table 7:** Agar disc diffusion to determine the capacity of the generated sheets to serve as antibacterial agents.

Sample	Components of sheet (%)				Inhibition zone (mm)		
	Shellac (5%)	Chitosan 2% (middle Mwt.)	Spinel (%)		*E. coli*	*B. mycoides*	*C. albicans*
			ZnAl_2_O_4_/C	CuAl_2_O_4_/C			
SSd	10	90	00	00	22	16	18
SSd1	10	90	0.05	00	18	17	18
SSd2	10	90	0.1	00	20	18	17
SSd3	10	90	00	0.05	17	19	20
SSd4	10	90	00	0.1	19	16	17

The dynamic shaking flask method was created for routine quality control and screening testing to overcome the constraints of standard antimicrobial test methodologies, such as ensuring proper contact of inoculums with treated surfaces. This variant of the shaking flask method is quicker to carry out since absorbance measurements are employed instead of the standard plating methodology. Traditional plating processes take days to yield data, but this method offers a measure of antimicrobial efficiency in hours. The antibacterial efficacy of the produced sheets was further evaluated using nutritional broth in a shake flask technique. To follow microbial development, the optical density of the media was measured over time. In comparison to the non-treated broth medium, Table 8 displays the percentage of microbial reduction following treatment by different produced sheets. The results reveal that the produced sheets are effective antibacterial agents against *E. coli, B. mycoides*, and *C. albicans*. The produced sheets had microbial growth inhibition percentages ranging from 85 to 98% (Table 8). The variations in growth density between the treated sheets and the control flasks are shown in Fig. 15.

**Table 8:** Shake flask experiment to demonstrate the antimicrobial activity of the prepared sheets.

Sample	Growth inhibition (%)		
	*E. coli*	*B. mycoides*	*C. albicans*
Control	0.00	0.00	0.00
SSd	90.14	85.83	93.90
SSd1	91.53	87.22	94.49
SSd2	94.03	88.75	97.32
SSd3	96.25	94.44	98.36
SSd4	97.22	95.69	98.51

300 mg of each sample/flask (50 ml medium), incubated for 4 h at 37 °C and 100 rpm.

**Fig. 16:**
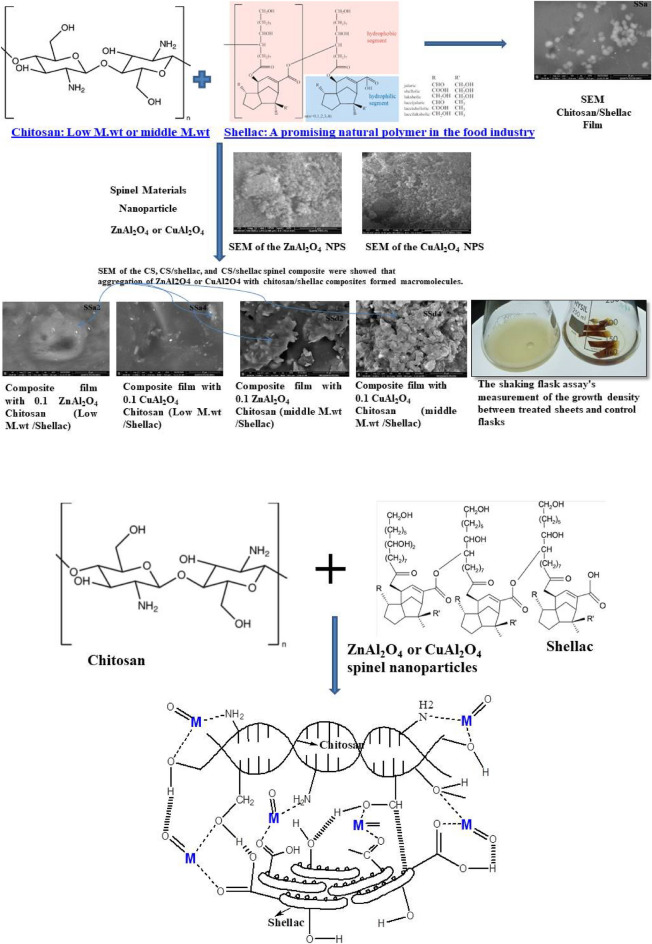
Diagram for the CS/shellac film formation and films treated with ZnAl_2_O_4_ and CuAl_2_O_4_ spinel nanoparticles and their suggested mechanism; M equal to Cu or Zn.

From the results obtained; by using the additive compounds to the prepared films we could get a good enhancement in the antimicrobial capacity of the films that are composed of the additives beside the well-known antimicrobial component, (i.e. chitosan). The shellac addition to the matrix of the film by a percentage of 10% and other spinel components helped in the compensation of a high-cost component (chitosan), and hence enhancing the economic value of the prepared films as well as its antimicrobial properties. Figure 16 shows a diagram for the CS/shellac film formation and films treated with ZnAl_2_O_4_ and CuAl_2_O_4_ spinel nanoparticles.

The food industry frequently uses shellac, a natural glue generated from insects, to glaze and treat citrus fruit and confectionery surfaces to avoid damage during storage^82,83^. It has a significant number of carbonyl and carboxyl groups in its chemical structure^82,84^.

Even though shellac has no antimicrobial properties, it is widely used as a non-toxic binder and coating material in food applications^82,85^. Shellac has been authorized by the FDA as GRAS, which means it can be used for indirect food contact. Shellac films also have remarkable adhesion to a variety of surfaces, as well as good stiffness, strength, and high gloss^83,85^. Chitosan is a multifunctional substance having antimicrobial action that has been proven. Antibacterial mechanisms have been postulated in three ways: (i) the ionic surface contact that causes wall cell leakage; (ii) the suppression of mRNA and protein synthesis caused by chitosan penetration into microorganism nuclei; and (iii) the development of an exterior barrier, chelating metals and causing microbial growth to be suppressed by limiting necessary nutrients. All the events are likely to happen at the same time, although at different intensities. The degree of acetylation (DA) and the molecular weight (MW) are other key variables in determining such activity. In general, the lower the MW and DA, the more effective it will be at inhibiting microbe growth and multiplication^86,87^.

As presented in the literature, when chitosan was employed separately, its antibacterial action was poor^88^. The biocomposite’s mechanism is to inhibit bacterial growth, which is possibly accomplished by forming cationic portions on the amine group and metal atom, which interact with the anionic portion on the microbial cell surface, and disturb bacterial growth by interfering with medium exchanges^88^.

Figure 16 shows a diagram for the CS/shellac film formation and films treated with ZnAl_2_O_4_ and CuAl_2_O_4_ spinel nanoparticles. An electrostatic interaction occurred between the positive charge of CS and the negative charge of shellac. Coordination complex (metal complex) was formed via coordination bonds between the lone pair of electrons of OH, NH and C=O groups of chitosan/shellac and the transition metal, Zn or Cu.

## Conclusion

New composite membranes, that show promise as food packaging materials, were synthesized from ZnAl_2_O_4_ and CuAl_2_O_4_ spinel nanoparticles and then combined with the natural chitosan and shellac polymers. With the use of FTIR spectra, the film structure was assessed in terms of component interactions. The percentage strain at maximum load, burst strength, Young’s modulus, and tensile strength were enhanced by 114–101%, 3.6–8.4, 103–119, and 179–153% for low and middle M.wt. CS, respectively, with the addition of 10% SH. At temperatures above 300 °C, the addition of ZnAl_2_O_4_ and CuAl_2_O_4_ composites provides greater thermal stability than shellac/CS composite. Additionally, it is evident from differential the DTG curves, that all composites mass loss rates clearly rise beyond 250 °C, demonstrating their comparatively superior high-temperature stabilities. When ZnAl_2_O_4_ and CuAl_2_O_4_ are added to shellac/CS (middle M.Wt.) the percentage of swelling decreased from 15.63 to 11.40% and the gel fraction of the film increased from 59.58 to 64.40% when 0.1% ZnAl_2_O_4_ is added. Similarly, when 0.05% CuAl_2_O_4_ is added, the swelling percentage decreases from 15.63 to 10.80% and the gel fraction increases from 59.58 to 65.82%. ZnAl_2_O_4_ (0.1%) and CuAl_2_O_4_ (0.05%) enhanced the gel fraction of the film from 59.58 to 64.40 and 65.82%, while lowered the swelling percentage from 15.63 to 11.40 and 10.80%, respectively. CuAl_2_O_4_-chitosan/shellac when compared to 0.1 additions, a composite with 0.05% spinel typically has better qualities. Using the shake flask method with nutrient broth and inhibition zone diameter as antimicrobial tools, the composite of 1:9 shellac/chitosan/0.05% of CuAl_2_O_4_ demonstrated the most Gram-positive antibacterial activity against *B. mycoides* (21 mm) and *C. albicans* (22 mm) as non-filamentous fungus. Therefore, these improvements make the composite films made of chitosan, shellac, ZnAl_2_O_4_, or CuAl_2_O_4_ a good substitute for making food packaging materials. Additionally, we can conclude that the shellac addition to the matrix of the film by a percentage of 10% and other spinel components helped in the compensation of a high-cost component (chitosan) and hence enhancing the mechanical qualities as well as low water permeability or hydrophobicity. As a result, they are also more stable in harsh environments like high pressure and temperature. The future scope of the work will be the study of the long-term stability or durability of the composite film under different environmental conditions, and the potential of composite films for real-world applications (e.g., food packaging) in terms of cost-effectiveness, scalability, and regulatory compliance, and then we will apply the best results on vegetables and fruits.

## Data Availability

All data generated or analysed during this study are included in this published article.
